# ATMAD: robust image analysis for Automatic Tissue MicroArray De-arraying

**DOI:** 10.1186/s12859-018-2111-8

**Published:** 2018-04-19

**Authors:** Hoai Nam Nguyen, Vincent Paveau, Cyril Cauchois, Charles Kervrann

**Affiliations:** 1 Inria Rennes - Bretagne Atlantique, Campus universitaire de Beaulieu, Rennes, 35042 France; 2 Innopsys, Parc d’Activités Activestre, Carbonne, 31390 France

**Keywords:** Tissue microarray, TMA de-arraying, Detection, Wavelet, Segmentation, Active contour, Deformation, Thin-plate spline

## Abstract

**Background:**

Over the last two decades, an innovative technology called Tissue Microarray (TMA), which combines multi-tissue and DNA microarray concepts, has been widely used in the field of histology. It consists of a collection of several (up to 1000 or more) tissue samples that are assembled onto a single support – typically a glass slide – according to a design grid (array) layout, in order to allow multiplex analysis by treating numerous samples under identical and standardized conditions. However, during the TMA manufacturing process, the sample positions can be highly distorted from the design grid due to the imprecision when assembling tissue samples and the deformation of the embedding waxes. Consequently, these distortions may lead to severe errors of (histological) assay results when the sample identities are mismatched between the design and its manufactured output. The development of a robust method for de-arraying TMA, which localizes and matches TMA samples with their design grid, is therefore crucial to overcome the bottleneck of this prominent technology.

**Results:**

In this paper, we propose an Automatic, fast and robust TMA De-arraying (ATMAD) approach dedicated to images acquired with brightfield and fluorescence microscopes (or scanners). First, tissue samples are localized in the large image by applying a locally adaptive thresholding on the isotropic wavelet transform of the input TMA image. To reduce false detections, a parametric shape model is considered for segmenting ellipse-shaped objects at each detected position. Segmented objects that do not meet the size and the roundness criteria are discarded from the list of tissue samples before being matched with the design grid. Sample matching is performed by estimating the TMA grid deformation under the thin-plate model. Finally, thanks to the estimated deformation, the true tissue samples that were preliminary rejected in the early image processing step are recognized by running a second segmentation step.

**Conclusions:**

We developed a novel de-arraying approach for TMA analysis. By combining wavelet-based detection, active contour segmentation, and thin-plate spline interpolation, our approach is able to handle TMA images with high dynamic, poor signal-to-noise ratio, complex background and non-linear deformation of TMA grid. In addition, the deformation estimation produces quantitative information to asset the manufacturing quality of TMAs.

**Electronic supplementary material:**

The online version of this article (10.1186/s12859-018-2111-8) contains supplementary material, which is available to authorized users.

## Background

### Tissue MicroArrays (TMA) history

The development of multi-tissue techniques was started at the mid-1980s in order to address the scarcity issue of diagnostic reagents and tissue samples. The pioneer work was contributed by Dr Battifora who introduced, in 1986, the multi-tumor “sausage” tissue block [1]. In this method, several rods of tissue, which were extracted from paraffin-embedded tissue blocks (or shortened as paraffin blocks), deparaffinized and rehydrated, were put together and reparaffinized after being tightly wrapped in small intestine of small mammals like a sausage. To avoid deparaffinization and reparaffinization procedures of Battifora’s “sausage” technique, in 1987, Wan et al. conceived the punching technique [2] which used 16-gauge needle for retrieving cylinders of tissue (also tissue cores) from paraffin blocks and arraying them in a recognizable pattern. Although Wan’s punching technique was a big footstep and was used in nearly all of today TMA techniques, its tissue pattern was not a grid one which is more structured and facilitates the identification of each tissue sample. The first multi-tissue grid pattern is described by Battifora and Mehta in their 1990’s paper under the name of “checkerboard tissue block” [3] in which tissue rods were manually aligned in a Cartesian coordinate system (checkerboard pattern). By combining the punching technique of Wan and the “checkerboard” concept of Battifora and Mehta, Kononen et al. invented in 1998 a machine for assembling efficiently and accurately extracted tissue cores in grid pattern [4]. The proposed technique called “tissue microarray” (TMA) became therefore popular and widely used in most pathological laboratories. In the last decade, different TMA techniques were developed to improve manufacturing process and minimize manufacturing cost [5–15], but all of them were based on Battifora’s, Wan’s and Kononen’s previous works. Since in most TMA techniques, extracted tissue samples have cylinder form, in the following, we use the terms “tissue cores” or “TMA cores” (or even more shorter cores) to refer TMA tissue samples.

### Challenges of TMA de-arraying

**Fig. 1:**
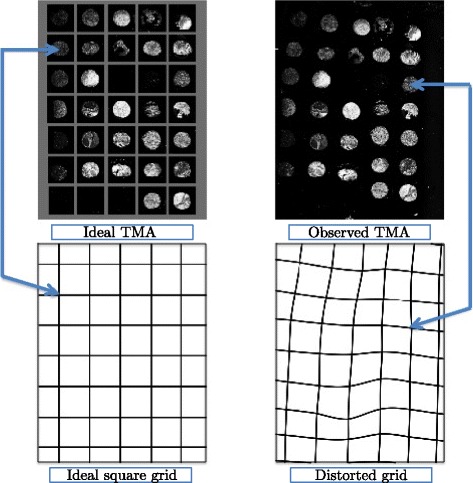
Deformation of the TMA grid. An ideal TMA (left top) has tissue cores perfectly aligned in vertical and horizontal directions with equal spacing according to a regular square grid (left bottom). The manufactured TMA (right top) is subjected to a non-linear deformation of the TMA grid resulting to a distorted grid (right bottom). We aim at de-arraying the observed TMA by estimating the deformation which transforms the ideal grid into the distorted grid

In a TMA, assembled tissue cores are collected from different donor blocks. It is thus highly important to matching them with their proper meta-data for further clinical or pathological analysis. To this end, grid pattern was conceived to ease the localization of each TMA cores. However, in spite of numerous technique improvements [12, 16], TMAs manufactured recently by manual or automated (semi-automated) machine are still subjected to the deformation of the design tissue grid due to bad positioning of the tissue cores with respect to the design. Another main source of deformation is the heat deformation of the paraffin waxes – commonly used in TMA techniques – when embedding tissue cores into recipient block. Sectioning paraffin-embedded tissue blocks with a microtome to produce multiple slides may also produce additional deformation. In fact, the design grid may suffer geometric transformations such as translation, rotation and shearing (linear or affine deformations) combined with dilatation, distortion and random perturbations (non-linear deformations). In addition, some fragile tissue cores may be lost or split into several fragmented parts, making more difficulties to recognize them. Figure 1 illustrates a typical image of TMA imaged in fluorescence. We can clearly observe that the ideal TMA grid which is a square grid is significantly distorted after the manufacturing process and the present tissue cores do not have a perfectly circular shape as expected. These problems need to be taken into account to develop robust de-arraying methods.

### State-of-art of TMA de-arraying methods

Closely similar to TMAs, DNA microarrays (also known as bio-chips) are constructed by spotting DNA probes by robots with high precision according to a grid pattern. Numerous gridding methods for microarrays were used to localize each DNA probes and find its row and column coordinates with respect to the design grid. This procedure is called “de-arraying”. Despite the similitude of these microarray concepts, existing “de-arraying” methods for microarrays are not adapted for TMAs because the grids are more highly deformed. Along with the commercialization of digital imaging devices for TMA analysis over the last decade, several methods for TMA “de-arraying” have been developed [17–22]. In general terms, a “de-arraying” approach consists in two steps: (i) segmentation and localization of assembled tissue cores; (ii) array coordinate (row and column coordinates) estimation of each core.

Firstly, for segmenting tissues, existing de-arraying methods usually assume that the histogram of a TMA image is bimodal. Under this assumption, these methods perform in general a thresholding by taking the local minimum between two highest peaks corresponding to the background and the foreground, of the image intensity histogram as global threshold. Various thresholding techniques were proposed from a simple thresholding as in [17] to more sophisticated methods such as the moment-preserving thresholding in [19], the automatic thresholding based on Savitsky-Golay filtered histogram in [20] or Otsu’s method used in [21, 22]. To improve the segmentation result, pre-processing like contrast enhancement transform [22] or template matching [19] was applied. Morphological operators were also used as post-processing for removing outliers in the thresholded map as in [17, 22]. However, this underlying assumption is not satisfied in case of images acquired from novel fluorescence device because of their complex background. Due to the nature of fluorescence imaging, pixels corresponding to irrelevant objects – such as dusts, glue and washing stains – in the background have often high intensities resulting as a high peak in the intensity histogram; in contrast, the intensities of pixels corresponding to tissue cores could be relatively lower. Hence, as a consequence, most of cores fail to be detected with a high threshold and there is a number of outliers corresponding to a low threshold value.

Secondly, for estimating row and column coordinates of each TMA cores, the methods mentioned above were generally based on distance and angle criteria to define the average spacing between the cores and the orientation of the observed grid. These criteria were derived simply from the distance between neighbor tissue cores [19], or from sophisticated measures such as the histogram of distance and angle [17] or the coefficients of the Hough transform [18] or even the Delaunay triangulation [22]. To deal with the case of missing tissue cores or the design of TMA grid in which some positions are left empty [16], linear or local bilinear interpolation were used as in [17, 22] for completing the grid. Whereas these methods yield satisfactory results for further pathological analysis, they can not produce quantitative information about the deformation of the TMA grid which is an indicator for evaluating the quality of the manufactured input TMA. For that reason, we address this issue and develop a de-arraying method which is able to provide quantitative information about the deformation. Our approach allows management of traceability and quality control of the whole TMA manufacturing process.

### Overview of the method

**Fig. 2:**
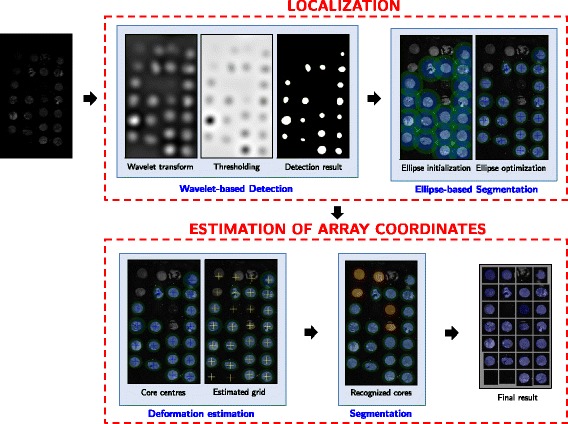
Overview of our TMA de-arraying approach. The proposed ATMAD approach consists in two steps : (i) tissue core localization; (ii) estimation of array coordinates of tissue cores. The localization step is performed by combining a fast wavelet-based detection and an ellipse-shaped active contour to produce accurate core positions for the second step. The second step is dedicated to the estimation of the deformation of the TMA grid. The objective is to refine the de-arraying result by providing additionally potential positions of tissue cores which were not recognized at the first step. The de-arraying result is presented as a regular array to facilitate the seeking of row and column coordinates of each core

In this paper, we propose a fast and efficient approach for automated TMA de-arraying with the emphasis on fluorescence TMA images and modeling of TMA grid deformation. The proposed approach called ATMAD is based on the following image processing operations: core detection, core segmentation and estimation of the grid deformation. For the tissue localization step of the de-arraying procedure, we combine the detection and segmentation tasks to produce reliable inputs for the second step – the computation of the array coordinate of each tissue core. This second step is performed by using the deformation estimation module followed by a segmentation task to refine the result. The outline of our approach is shown in Fig. 2 which describes the two steps of the de-arraying procedure and the combination of the three image processing operations.

The “detection” operation (*i.e.* the detection) is based on a wavelet approach. In order to process images having large dynamic range, complex background and high noise level such as fluorescence images, we compute a stationary wavelet transform of the input TMA image at an appropriate scale to the tissue size – the average tissue core radius given by the manufacturer. By choosing the mother wavelet as a difference of Gaussians, we can deduce the closed-form expression of the wavelet atom at any desired scale and use it to perform directly the wavelet decomposition. Our technique is faster and more accurate than the well-known “à trous” algorithm [23]. The wavelet transform map is then locally thresholded to spatially adapt to the contrast between the foreground – corresponding to TMA cores – and the inhomogeneous background. The position of potential tissue cores is defined as the center of the connected components in the thresholded wavelet transform map.

To delineate the boundary of each tissue core and improve detection result, an ellipse-shaped active contour [24] is used for segmenting the detected object at each position obtained from previous step. The segmented objects, which are too large or too small than the given average size of tissue sample or too elongated, will be considered as false detection and be discarded from the list of potential positions. This removal is essential to discard potential outliers and enhance the reliability of the input for the estimation of row and column coordinates of TMA cores.

Instead of estimating directly the row and column coordinates of each core from the position list, we approximate the deformation of the TMA grid using the thin-plate model. In fact, the deformed grid is the image (in the sense of set theory) of the regular grid of design by the deformation. Given the deformation at some arbitrary points of the grid, the thin-plate interpolation allows to estimate it at other points [25]. The more points we have known, the more precisely we estimate the deformation. Once the deformation is approximated, the computation of row and column coordinates of each tissue core is therefore straightforward. By reformulating as an approximation problem and solving it iteratively, our method is robust to high non-linear deformations which were observed in most real TMA images. Moreover, according to the thin-plate model, the approximation yields information such as the average translation, the rotation angle, the bending energies along the horizontal and vertical axes, etc. These information are useful to assess the quality of the manufactured TMAs.

The remainder of this paper is organized as follows. In the next section, we describe the de-arraying approach including a technical presentation of the “detection, “segmentation”, “deformation estimation” tasks. We also figure out how the proposed approach is adapted for TMA images acquired with brightfield microscopes. In “Results and discussion” section, we present the experimental results obtained from simulated and real data. Finally, the last section gathers the conclusions drawn from this research and details the future work.

## Methods

**Fig. 3:**
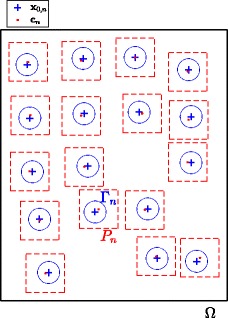
Illustration of core positions and notations. The image *u* is defined on a rectangular domain *Ω* (shown in black rectangle). For each detected position **c**_*n*_ (red small dots), a patch *P*_*n*_ (red dashed squares) centered at **c**_*n*_ is extracted. The ellipse *Γ*_*n*_ (light blue ellipses) with center **x**_0,*n*_ (blue crosses) is optimized to fit the object of interest which is located inside the patch *P*_*n*_

In our approach, the estimation of core positions on the input TMA image is subsequently refined in successive tasks by considering different image domains (i.e. patches or regions) in the input original image. Such a strategy allows not only to avoid unnecessary processing on non-content regions but also to reduce the acquisition time, storage and processing time of high resolution data. To distinguish the inputs and outputs of each task and facilitate the comprehension of the technical details, we present in Fig. 3 a diagram which illustrates a few notations which will be used throughout the paper.

### TMA core detection

The detection of approximately circular TMA cores can be performed by spot detection algorithms. Spot detection is a well-known topic in image processing (see [26] for a recent review). Over past decades, number of spot detection methods have been proposed [27–31]. To produce satisfactory results, most of these methods require fine adjustment of a critical parameter: the detection scale corresponding to the size of the objects of interest. Automatic selection of the detection scale is a challenging problem since the objects of interest may have different sizes or they may have the same size as the irrelevant objects in the background. Few methods of automatic scale selection [32–34] have been proposed recently. However, in the context of tissue microarrays, the diameter of assembled TMA cores is defined by the size of the needle used for extracting cores from paraffin tissue blocks. The determination of the scale parameter used for spot detection is straightforward from this measure which is often given by the manufacturer. We propose here a fast algorithm for tissue core detection by performing directly the wavelet decomposition at the appropriate aforementioned scale and computing a locally-adaptive threshold of the wavelet coefficients.

### Pre-processing

Wavelet-based detection techniques are known to be robust to non-stationary noises like Poisson noise or mixed-Poisson-Gaussian noise as in TMA images, acquired by brightfield or fluorescence. Pre-processing operations such as image denoising or variance stabilization transform are thus not mandatory. However, our algorithm is primarily designed for detecting bright spots over a dark background, and especially adapted for fluorescence images. brightfield TMA images, in which the tissue cores are darker than the background, are first inverted before further processing.

### Scale selection

Nowadays, standard TMAs are manufactured with a diameter of tissue core typically from 0.6 to 1.5 mm. Given an imaging resolution (pixel size), the optimal scale of wavelet decomposition can be determined according to the core radius. If we denote *r*_*core*_ the expected average radius (in pixels) of TMA cores, the optimal scale index ${\hat {\jmath }}$ of the wavelet decomposition that best fits the size of TMA cores is defined as:

1 $$ \hat{\jmath} = \underset{j \in \mathbb{N}^{*}}{\text{argmin}} \left| {r}_{core} - 2^{j-1}\sigma_{1} \right| \,  $$

where *σ*_1_ is selected according to the pixel size.

### Fast isotropic wavelet decomposition

In contrast to multiresolution approaches, our detection method requires only the wavelet decomposition at the appropriate selected scale. To compute the decomposition at a desired scale, usual wavelet transform techniques perform a sequence of successive convolutions which are used for computing iteratively the decomposition from the smallest scale to the coarsest scale. These techniques are time consuming when dealing with large images and high number of scales. Instead, to address to this computational issue, we build a dyadic isotropic wavelet frame $\left \lbrace \psi _{j} \right \rbrace _{j \geq 1}$ by choosing the scaling function *ϕ*_*j*_ as a Gaussian function whose variance $v_{j}^{2}$ is a function of scale $j \in \{1, \ldots, j_{\max }\}$ and $j_{\max }$ is the maximum index of the highest scale:

2 $$ \phi_{j}(\mathbf{x}) = G_{v_{j}}(\mathbf{x}) = \frac{1}{{2\pi v_{j}}}\exp \left(-\frac{\left\| \mathbf{x} \right\|_{2}^{2}}{2v_{j}^{2}} \right),  $$

where ∥·∥_2_ denotes the Euclidean norm, $\mathbf {x} \in \Omega \subset \mathbb {R}^{2} $ is the pixel location in the rectangular domain *Ω* and

3 $$ v_{j}^{2} = \sum_{k = 1}^{j} \sigma_{k}^{2} = \sum_{k = 1}^{j} 4^{k-1}\sigma_{1}^{2} = \sigma_{j}^{2} + v_{j-1}^{2}  $$

with *σ*_*k*_=2^*k*−1^ *σ*_1_. Thanks to the semi-group property of Gaussian functions, the relationship between the scaling functions at subsequent scales can be expressed as:

4 $$\begin{array}{@{}rcl@{}} \phi_{j}(\mathbf{x}) &=& G_{\sqrt{\sigma_{j}^{2} + v^{2}_{j-1}}} (\mathbf{x})\\ &=& G_{\sigma_{j}} \star G_{v_{j-1}}(\mathbf{x}) = G_{\sigma_{j}} \star \phi_{j-1}(\mathbf{x}),  \end{array} $$

where ⋆ denotes the convolution operator. Therefore, the wavelet decomposition *Ψ*_*j*_*u* of $u: \Omega \subset \mathbb {R}^{2} \rightarrow \mathbb {R} $ at the scale $j \in \{1, \ldots, j_{\max }\}$ is obtained by convolution of *u* with the wavelet atom *ψ*_*j*_ as:

$$\begin{array}{@{}rcl@{}} \Psi_{j} {u}(\mathbf{x}) = \psi_{j} \star {u}(\mathbf{x}) & = &\left(\phi_{j - 1} - \phi_{j} \right) \star {u}(\mathbf{x})  \\ & = & (G_{v_{j-1}} - G_{v_{j}}) \star {u}(\mathbf{x}),  \end{array} $$

with the conventions $v_{0}^{2} = 0$ and *G*_0_(·)=*δ*(·) (Dirac delta function). For more technical details on the proposed wavelet frame and the wavelet decomposition and reconstruction algorithms, please refer to the Additional file 1.

### Locally-adaptive thresholding

While the wavelet decomposition plays the role of a filtering which reduces the noise and enhances the objects of interest, a common way to detect objects is to threshold the filtered image – the wavelet decomposition of the input TMA image in our case. As depicted in [32], a global threshold is not appropriate to handle complex situations, especially when dealing with images acquired in fluorescence context because of their inhomogeneous background. To overcome this difficulty, we propose to define an adaptive threshold according to the local distribution of the wavelet decomposition $\Psi _{\hat {\jmath }}{u}$ previously computed. Accordingly, we consider the following statistical test at each point **x** of the TMA image *u*:

$$\begin{array}{@{}rcl@{}} \left\lbrace \!\! \begin{array}{ll} \mathcal{H}_{0}: & \mathbf{x}\, \text{belongs to the background,} \\ \mathcal{H}_{1}: & \mathbf{x}\, \text{corresponds to tissue core (foreground).} \end{array} \right. \end{array} $$

Pixels corresponding to tissue cores have strong positive responses in the wavelet decomposition. Under the null hypothesis $\mathcal {H}_{0}$, the wavelet coefficient $\Psi _{\hat {\jmath }}{u}(\mathbf {x})$, which follows the local distribution of the wavelet-decomposed-image background with mean *μ*(**x**) and variance *ν*^2^(**x**), is lower than a certain value *τ*(**x**). Let $\mathbb {P} \left (\Psi _{\hat {\jmath }}{u}(\mathbf {x}) < \tau (\mathbf {x}) \right)$ be the probability for a pixel **x** to be classified as “background” class. The threshold *τ*(**x**) is used to control the number of misclassification. Given a probability of false alarm $p_{_{\text {FA}}} > 0$, the corresponding threshold $\tau _{_{\text {FA}}}$ is selected such that the misclassification probability $\mathbb {P} \left (\Psi _{\hat {\jmath }} {u}(\mathbf {x}) \geq \tau _{_{\text {FA}}}(\mathbf {x}) \right)$ is lower than $p_{_{\text {FA}}}$. By applying the conventional probabilistic Tchebychev’s inequality, we get, ∀*κ*(**x**)>0:

5 $$ \mathbb{P} \left(\left| \Psi_{\hat{\jmath}} {u}(\mathbf{x}) - \mu(\mathbf{x}) \right| \geq \kappa(\mathbf{x}) \right) \leq \frac{\nu^{2}(\mathbf{x})}{\kappa^{2}(\mathbf{x})}.  $$

It follows that

$$\mathbb{P} \left(\Psi_{\hat{\jmath}} {u}(\mathbf{x}) \geq \mu(\mathbf{x}) + \kappa(\mathbf{x}) \right) \leq \mathbb{P} \left(\left|\Psi_{\hat{\jmath}} {u}(\mathbf{x}) - \mu(\mathbf{x}) \right| \geq \kappa(\mathbf{x}) \right). $$

Now, let us define $\tau _{_{\text {FA}}}(\mathbf {x}) = \mu (\mathbf {x}) + \kappa (\mathbf {x})$ and assume $\left ({\nu ^{2}(\mathbf {x})}/{\kappa ^{2}(\mathbf {x})}\right) \leq p_{_{\text {FA}}}$ such that $\mathbb {P} \left (\Psi _{\hat {\jmath }} {u}(\mathbf {x}) \geq \tau _{_{\text {FA}}}(\mathbf {x}) \right) \leq p_{_{\text {FA}}}$. Finally,

6 $$ \tau_{_{\text{FA}}}(\mathbf{x}) \geq \mu(\mathbf{x}) + \frac{\nu(\mathbf{x})}{\sqrt{p_{_{\text{FA}}}}}  $$

and the adaptive threshold $\tau _{_{\text {FA}}}(\mathbf {x})$ is controlled by the *p*-value inferred from the significance level *α* of the test, and set by the user. If $p_{_{\text {FA}}} < \alpha $, this suggests that the null hypothesis (i.e. a pixel **x** is classified as a “background” pixel) may be rejected. In practice, one typically sets $p_{_{\text {FA}}} = 0.05$ (or 0.01) which corresponds to a significance level *α*=5*%* (or 1% respectively).

**Fig. 4:**
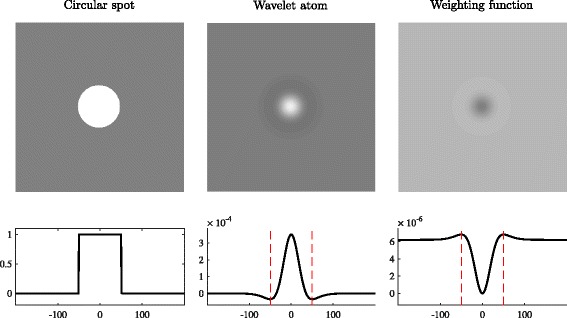
Wavelet atom and corresponding weighting function used for estimating the local distribution wavelet transform of a circular spot image. From left to right : the image of a circular spot, its wavelet atom at the appropriate scale and its corresponding weighting function on the top row;and their radial profile on the bottom row (red dashed lines delineate the radius of the spot)

To determine the threshold $\tau _{_{\text {FA}}}(\mathbf {x})$, the local mean *μ*(**x**) and the local variance *ν*^2^(**x**) of the image background on the wavelet decomposition $\Psi _{\hat {\jmath }}{u}$ are required. However, prior knowledge about the image background distribution is unfortunately not available in most cases. We consider thus empirical estimations of *μ* and *ν*^2^ at each point **x** from $\Psi _{\hat {\jmath }}{u}$:

7 $$\begin{array}{@{}rcl@{}} \hat{\mu}(\mathbf{x}) &=& g \star \Psi_{\hat{\jmath}}{u}(\mathbf{x}) \, \end{array} $$

8 $$\begin{array}{@{}rcl@{}} \hat{\nu}^{2}(\mathbf{x}) &=& g \star \left(\Psi_{\hat{\jmath}}{u}\right)^{2}\!(\mathbf{x}) - \hat{\mu}^{2}(\mathbf{x}) \,  \end{array} $$

where *g*(·) is a weighting positive function (i.e. ∥*g*(·)∥_1_=1, ∥·∥_1_ is the *L*_1_ norm and *g*(**x**)≥0,∀**x**∈*Ω*) mainly used to avoid the estimation of the background distribution statistics being biased from coefficients corresponding to the foreground. By construction, $\hat {\mu }(\mathbf {x})$ and $\hat {\nu }^{2}(\mathbf {x})$ are weighted estimators derived from $\Psi _{\hat {\jmath }}{u}$ which is a filtered version of *u* by the band-pass filter $\psi _{\hat {\jmath }}$ in order to enhance the objects of radius *r*_*core*_. It is thus convenient to define the weighting function *g* according to the wavelet atom $\psi _{\hat {\jmath }}$. By using an affine transform which implies the positivity and the normalization conditions, we propose a candidate for *g*(·) as follows :

9 $$ \hat{g}(\mathbf{x}) = \frac{\ -\psi_{\hat{\jmath}}(\mathbf{x}) + \sup \psi_{\hat{\jmath}} \ }{\ \left\| -\psi_{\hat{\jmath}} + \sup \psi_{\hat{\jmath}} \right\|_{1} \ } \,  $$

where $\sup \psi _{\hat {\jmath }} = \| \psi _{\hat {\jmath }} \|_{\infty }$ denotes the supremum ($L_{\infty }$ norm) of $\psi _{\hat {\jmath }}$ and $\left \| -\psi _{\hat {\jmath }} + \sup \psi _{\hat {\jmath }} \right \|_{1}$ is the normalization factor to ensure $\|\hat {g}(\cdot)\|_{1} = 1$. The choice of this candidate is clarified in Fig. 4 showing the wavelet atom and its derived weighting function according to a given circular spot. The proposed weighting which is constructed from the wavelet atom has the same size of the considered spot and has a hollow shape at the center (see right column in Fig. 4). This specific shape allows to reduce the impact of high wavelet coefficients corresponding to foreground pixels on the estimation of the background statistics.

By substituting the empirical estimators to *μ*(**x**) and *ν*^2^(**x**), we obtain the estimated detection threshold:

10 $$ \hat{\tau}_{_{\text{FA}}}(\mathbf{x}) = \hat{\mu}(\mathbf{x}) + \frac{\hat{\nu}(\mathbf{x})}{\sqrt{p_{_{\text{FA}}}}} \ .  $$

Thresholding the wavelet decomposition $\Psi _{\hat {\jmath }}{u}$ with respect to $\hat {\tau }_{_{\text {FA}}}$ results in a binary image $I_{_{\text {FA}}}: \Omega \rightarrow \{0,1\}$:

11 $$\begin{array}{@{}rcl@{}}I_{_{\text{FA}}} (\mathbf{x}) = \left\{ \begin{array}{ll} 1 & \text{if}~~ \Psi_{\hat{\jmath}}{u} (\mathbf{x}) \geq \tau_{_{\text{FA}}}(\mathbf{x})\\ 0 & \text{otherwise} \end{array} \right. \end{array} $$

where each connected component in $I_{_{\text {FA}}}$ represents a region which is potentially a tissue core of the TMA image. The gravity centers of these regions (or detection position) will be used as inputs for estimating the array coordinates of TMA cores. However, the detection reliability has a great impact on the de-arraying outcome: few false detections may lead to severely inaccurate results. Removing false detections (i.e. outliers) is then crucial. To this end, the size of detected regions seems to be a relevant criterion since the core size is given in most cases by the TMA manufacturer. Although, due to the complexity of backgrounds, it may be highly different from the true core size. Instead of exploiting the imprecise information derived from the binary detection map $I_{_{\text {FA}}}$, we perform an active-contour-based segmentation to delineate the objects at each detected position. Also, we re-use the segmentation results to confirm and improve the preliminary detection results.

### Segmentation of TMA cores

As depicted in previous section, the detection binary image $I_{_{\text {FA}}}$ does not allow us to accurately determine the size of detected objects. Active contours [35] are typically well appropriate in our context since they can evolve to closely delineate the object borders and thus yield an estimation of the TMA core size. The family of parametric active contours presented below will help to refine the detected position and the size of TMA cores and eventually to determine the orientation of the potential core if it was deformed during the manufacturing process.

Since the seminal paper of Kass, Witkin, and Terzopoulos, active contour models (or snakes) [35] have been successfully used to detect discontinuities, detect objects of interest or segment images, especially in bioimaging [36]). General purpose closed contours are generally controlled by elastic forces based on local curvature and image based potentials [35, 37–39]. The curve evolves from its initial starting position towards the target object. The optimization of the underlying energy functional is traditionally performed using variational principles and finite differences techniques, which needs an appropriate initialization to converge to a relevant solution. At the end of the nineties and beginning of the 2000’s, geodesic active contours [40] based on the theory of surfaces evolution and geometric flows have been introduced to segment an arbitrary number of highly complex objects in the image. In our TMA context, the 2D shapes of tissue cores can be actually well estimated by ellipse-shaped active contours which belong to the family of parametric deformable templates.

Application-tailored parametrized templates introduced by Yuille et al. [41] were proposed in cases where strong a priori knowledge about the shape being analyzed is available (e.g. eyes or lips in human faces [41]). The models are hand-built using simple parametrized 2D geometric representations. Another line of research focused on models of random deformations for a given initial shape (deformable template). Grenander et al. [42, 43] obtained the first promising results in image segmentation by considering statistical deformable models which describe the statistics of local deformations applied to an original template. Markov models and Monte-Carlo techniques have been introduced in this context to derive optimal random deformations estimates from image data [42–46]. In the approach initially proposed by Cootes et al. [47] and successfully applied to object tracking [45], the shape structure and the parameters describing its deformations are learned from a training set of representative shapes. Meanwhile, Staib and Duncan [48] proposed to combine parametric snakes (B-splines) to the standard decomposition on a Fourier basis to analyze deformable biomedical structures. All these methods are generally robust to noise but computationally demanding if stochastic iterative procedures are used to conduct the minimization and no initial guess close to the optimal solution is provided. Very recently “snakescules” [49] combined to fast algorithms and Markov point process [50] have been proposed along the same philosophy but dedicated to the detection of cells or nuclei in fluorescence microscopy images.

**Fig. 5:**
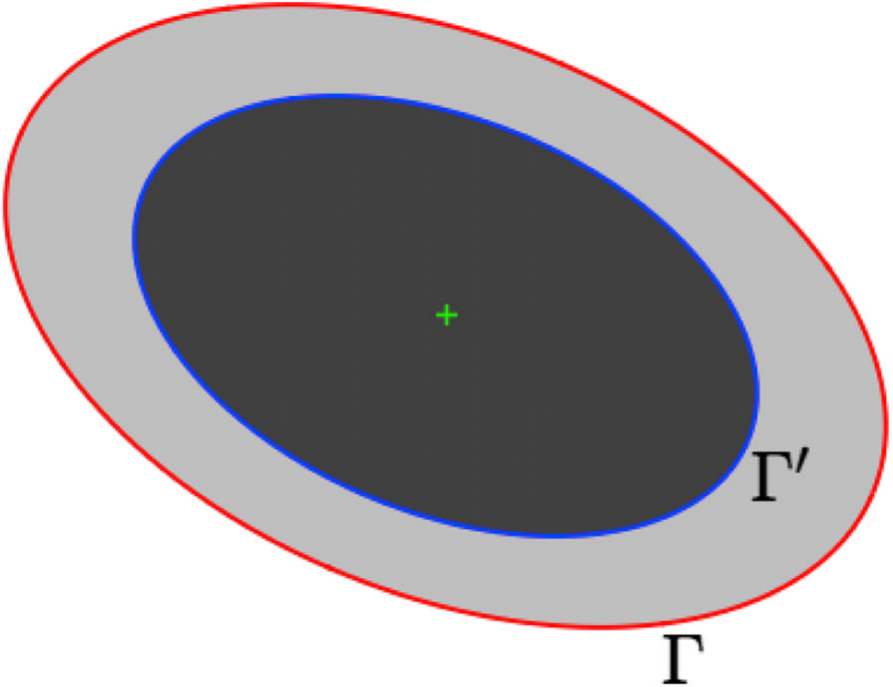
Pair of concentric and coaxial ellipses. The outer ellipse *Γ* (red curve) has an area twice larger than the inner ellipse *Γ*^′^ (blue curve). These ellipses determine two domains of the same area : an elliptical outer ring (shown in light gray) and an elliptical inner core (dark gray)

Finally, the ellipse fitting concept has been furthemore introduced by Thévenaz et al. as an extension of the simple circle-shaped active contour [49] which can be defined just by two points [24]. As a consequence, a triplet of points is necessary to parametrize the ellipse-shaped version. However, this parametrization which has an extra degree of freedom increases the complexity of the model and makes the optimization of ellipse parameters more challenging when compared to the circle-shaped model. To overcome these difficulties, an alternative way was proposed in [51]: the ellipses are configured by their center, their axes and the angle between their major axis and the horizontal. Under this configuration, the cost function introduced in [24], and defined below (see Eq. (12)) as the contrast between the core and the ring defined by the pair of ellipses (see Fig. 5) – and the derivatives with respect to the ellipse parameters could be calculated efficiently by using the Green’s theorem [48]. Nevertheless, the Green’s theorem cannot be applied with no error in the discrete setting and digitized images. In order to handle properly the ellipse parametrization described in [51] instead of [24] in the discrete setting, we propose a pixel-based smooth approximation of the underlying cost energy functional. Our approximation allows us to calculate properly the derivatives of the cost function with respect to the ellipse parameters and is not based on the Green’s theorem also used in [48] for energy minimization.

### Definition of the ellipse-based energy

More formally, let *Γ* be the outer ellipse with parameters $\left \lbrace \mathbf {x}_{0}, a, b, \theta \right \rbrace $ where $\mathbf {x}_{0} = \left (x_{0}, y_{0} \right)$ is the center, *a* and *b* are the semi major and minor axes respectively, and *θ* is the angle of rotation. The inner ellipse ${\Gamma }^{\prime }$ is defined as a concentric and coaxial ellipse of *Γ* such the latter has an area (denoted |*Γ*|) twice larger than the former: $|{\Gamma }| = 2|{\Gamma }^{\prime }|$ (see Fig. 5). The factor 2 ensures that the area of the elliptical outer ring is equal to the area of the elliptical inner core. Let us consider a rectangular image patch *P* containing a potential TMA core associated to a connected component estimated by the detection method in the early stage. The ellipse energy (or cost function) is defined as a normalized image contrast between the two domains ${\Gamma }^{\prime }\subset {\Gamma } \subset P$ where *P* is a rectangular domain in the image domain *Ω* which contains a single TMA core [24, 52]:

12 $$\begin{array}{@{}rcl@{}} J(u, {\Gamma}) & = & \frac{1}{ab} \left(\int_{{\Gamma} \backslash {\Gamma}^{\prime}} u(\mathbf{x}) \ d\mathbf{x} - \int_{{\Gamma}^{\prime}} {u}(\mathbf{x}) \ d\mathbf{x}\right)~~~~~~ \\ & =& \frac{1}{ab} \left(\int_{{\Gamma}} {u}(\mathbf{x}) \ d\mathbf{x} - 2\int_{{\Gamma}^{\prime}} {u}(\mathbf{x}) \ d\mathbf{x}\right).  \end{array} $$

To handle discrete images, the continuous image *u* defined in (12) can be replaced by its sampled version as follows: 
13

where $u\left [\mathbf {x}\right ]$ is the discrete sample of *u*(**x**) and  denotes the set indicator function such as  if **x**∈*Γ* and 0 otherwise. However, there are two major drawbacks while considering this energy function. Firstly, the calculation of the energy gradient is not trivial in the discrete setting since the indicator functions in (13) are piecewise constant which are not differentiable at some points. Secondly, due to sampling effect, brutal switch of the membership of some points from a domain to another may happen just with an infinitesimal change in the ellipse parameters, giving rise to severe numerical instabilities. Smooth approximations of the underlying piecewise constant functions is recommended to overcome both discontinuity and sampling problems. The calculations of partial derivatives of the energy functional is facilitated if we can define a fuzzy membership to avoid abrupt domain switches (see Fig. 8a and b). Our goal is then to build an approximation which favors the computation of the partial derivative of the energy with respect to each ellipse parameter as much as possible. First, we consider the following quadratic form:

14 $$\begin{array}{@{}rcl@{}} \left\|\mathbf{x} \right\|_{{\Gamma}}^{2} = \left\| \left[ \begin{array}{cc} a^{-1} & 0 \\ 0 & b^{-1} \end{array} \right] \left[ \begin{array}{cc} \cos\theta & \sin\theta \\ -\sin\theta & \cos\theta \end{array} \right] \mathbf{x} \ \right\|_{2}^{2}. \end{array} $$

**Fig. 6:**
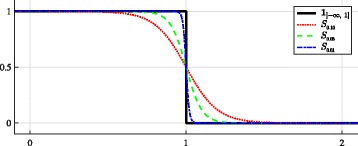
Approximation of the indicator function by logistic curves. The smaller *ε*, the closer the S-shaped curve *S*_*ε*_ approaches the graph of

**Fig. 7:**
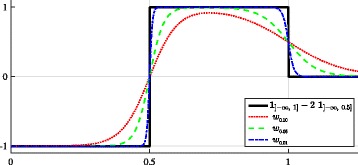
The weights *w*_*ε*_. $w_{\epsilon }\left (\left \| \mathbf {x} - \mathbf {x}_{0}\right \|_{\Gamma }^{2} \right)$ approximates  whose the normalized radial profile is presented by the graph of the piecewise constant function

For a given point **x**, $\left \| \mathbf {x} - {\mathbf {x}_0} \right \|_{{{\Gamma }}}$ is a normalized metric between **x** and the ellipse center **x**_0_ induced by the geometry of the ellipse *Γ*. A pixel **x** belongs to the interior of the ellipse *Γ* if and only if $ \left \| \mathbf {x} - {\mathbf {x}_0} \right \|_{{{\Gamma }}}^{2} \leq 1$ since $\|\mathbf {x} - {\mathbf {x}_0} \|_{{{\Gamma }}}^{2}$ is always positive. The term  can be then expressed by a function of $\left \| \mathbf {x} - {\mathbf {x}_0} \right \|_{{{\Gamma }}}$ as . Moreover, we need to find a smooth function which closely approximates  as investigated in [24, 38] and has simple derivative. We realized that the graph of  looks similar to the $C^{\infty }$ S-shaped logistic curve whose the derivative is easy to compute. Let us consider therefore the following logistic function: 
15

where *ε*>0 controls the steepness of the curve (see the plot of *t*↦*S*_*ε*_(*t*) in Fig. 6 for several values of *ε*). The smaller *ε*, the closer the curve *S*_*ε*_ approaches the graph of the indicator function . Thanks to the property of logistic functions, the derivative of *S*_*ε*_ can be easily computed as $S_{\epsilon }'(t)\,=\,-\epsilon ^{-1}\!S_{\epsilon }(t) \left (1 - S_{\epsilon }(t)\right)$. Finally, the energy functional has the following form [24]:

16 $$  J(u, {\Gamma}) = \frac{1}{ab} {\sum_{\mathbf{x} \in P \cap \mathbb{Z}^{2}}} w_{\epsilon} \left(\|\mathbf{x} - {\mathbf{x}_0} \|_{{{\Gamma}}}^{2}\right)\, {u}\left[\mathbf{x}\right] \,  $$

with $w_{\epsilon }(t) = S_{\epsilon }(t) - 2S_{\epsilon }(2t) = \frac {1}{1 + e^{\frac {t-1}{\epsilon }}} - \frac {2}{1 + e^{\frac {2t-1}{\epsilon }}}$. For illustration, we present in Fig. 7 the plot of *w*_*ε*_ whose the term $w_{\epsilon }\left (\| \mathbf {x} - {\mathbf {x}_0} \|_{{\Gamma }}^{2}\right)$ is nothing else than a smooth approximation of the piecewise constant function . These weights are very similar to those described in [38] and based on the $\arctan $ function.

### Calculation of partial derivatives

**Fig. 8:**
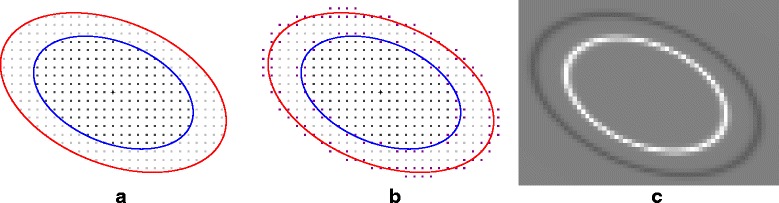
Inner and outer domain membership under discrete setting. Points in the inner core are marked by dark gray squares and those in the outer ring are marked by lighter gray squares. From left to right : **a** abrupt domain switch for points in the neighbor of ellipse boundaries (red and blue curves); **b** fuzzy membership with transition zones (marked by purple squares); and **c** first order derivative of the function *w*_*ε*_ (zero values are shown in gray)

By applying the derivation rules of composite functions, the partial derivatives of the energy with respect to each ellipse parameter $\left \lbrace \mathbf {x}_{0}, a, b, \theta \right \rbrace $ are given by:

$$\begin{array}{@{}rcl@{}} \left\{ \begin{array}{ll} \!\!\!\frac{\partial J(u, {{\Gamma}})}{\partial x_{0}} & \!\!\!\!\!\! = \frac{1}{ab} \sum_{\mathbf{x}} {u}[\mathbf{x}] w_{\epsilon}' \left(\|\mathbf{x} - {\mathbf{x}_0} \|_{{{\Gamma}}}^{2} \right) \frac{\partial \|\mathbf{x} - {\mathbf{x}_0} \|_{{{\Gamma}}}^{2}}{\partial x_{0}}, \\ \!\!\!\frac{\partial J(u, {{\Gamma}})}{\partial y_{0}} & \!\!\!\!\!\! = \frac{1}{ab} \sum_{\mathbf{x}} u[\mathbf{x}] w_{\epsilon}' \left(\|\mathbf{x} - {\mathbf{x}_0} \|_{{{\Gamma}}}^{2} \right) \frac{\partial \|\mathbf{x} - {\mathbf{x}_0} \|_{{{\Gamma}}}^{2}}{\partial y_{0}}, \\ \!\!\!\frac{\partial J(u, {{\Gamma}})}{\partial \theta} & \!\!\!\!\!\! = \frac{1}{ab} \sum_{\mathbf{x}} u [\mathbf{x}] w_{\epsilon}' \left(\|\mathbf{x} - {\mathbf{x}_0} \|_{{{\Gamma}}}^{2} \right) \frac{\partial \|\mathbf{x} - {\mathbf{x}_0} \|_{{{\Gamma}}}^{2}}{\partial \theta}, \\ \!\!\!\frac{\partial J(u, {{\Gamma}})}{\partial a} & \!\!\!\!\!\! = \frac{1}{ab} \sum_{\mathbf{x}} u [\mathbf{x}] w_{\epsilon}' \left(\|\mathbf{x} - {\mathbf{x}_0} \|_{{{\Gamma}}}^{2} \right) \frac{\partial \|\mathbf{x} - {\mathbf{x}_0} \|_{{{\Gamma}}}^{2}}{\partial a} - \frac{J(u, {{\Gamma}})}{a},~~~ \\ \!\!\!\frac{\partial J(u, {{\Gamma}})}{\partial b} & \!\!\!\!\!\! = \frac{1}{ab} \sum_{\mathbf{x}} u[\mathbf{x}] w_{\epsilon}' \left(\|\mathbf{x} - {\mathbf{x}_0} \|_{{{\Gamma}}}^{2} \right) \frac{\partial \|\mathbf{x} - {\mathbf{x}_0} \|_{{{\Gamma}}}^{2}}{\partial b} - \frac{J(u, {{\Gamma}})}{b},~~~~ \end{array} \right.  \end{array} $$

$$ \begin{array}{l l} \text{where}~ w_{\epsilon}'(t) & = \frac{4S_{\epsilon}(2t)\left(1 - S_{\epsilon}(2t) \!\right) - S_{\epsilon}(t)\left(1 - S_{\epsilon}(t)\right)}{\epsilon} \\ \\ & = \frac{1}{\epsilon} \left[ \frac{4e^{\frac{2t-1}{\epsilon}}}{\left(1 + e^{\frac{2t-1}{\epsilon}} \right)^{2}} - \frac{e^{\frac{t-1}{\epsilon}}}{\left(1 + e^{\frac{t-1}{\epsilon}} \right)^{2}}\right] \end{array} $$

and the calculation of partial derivatives of $\|\mathbf {x} - {\mathbf {x}_0} \|_{{{\Gamma }}}^{2}$ are detailed in the Additional file 1. As depicted in Fig. 8c, for a given parametrization {**x**_0_,*a,b*,*θ*}, the term $w_{\epsilon }'\left (\| \mathbf {x} - {\mathbf {x}_0} \|_{{{\Gamma }}}^{2} \right)$ vanishes for most of points **x**. Thus, the computation of the partial derivatives *J*(*u*,*Γ*) takes account only few points near the ellipse boundaries where $w_{\epsilon }'\left (\|. - {\mathbf {x}_0} \|_{{{\Gamma }}}^{2} \right)$ is non-zero. Our smooth approximation which is adapted for discrete images produces similar expressions of the partial derivatives of the ellipse energy when comparing with those described in [51] for continuous images. It can be viewed as the expression of the Green’s theorem in the discrete setting and an alternative to the optimization presented in [44].

### Multi-ellipse segmentation for multi-tissue core analysis

Let {**c**_*n*_}_1≤*n*≤*N*_ be the centroids of the connected components of the binary detection map $I_{_{\text {FA}}}$. In the original image *u*, we extract a rectangular patch *P*_*n*_ centered at **c**_*n*_ with a radius *ρ* larger than the given tissue core radius *r*_*core*_ (for example, *ρ*=2*r*_*core*_). Let us define

17 $$ \Pi_{\rho, \mathbf{c}_{n}} u = \left\lbrace {u}\left[\mathbf{x}\right], \ \left\| \mathbf{x} - \mathbf{c}_{n} \right\|_{\infty} \leq \rho \right\rbrace,  $$

where **x**=(*x,y*)∈*P*_*n*_, $\|\mathbf {x}\|_{\infty } = \sup (|x|,|y|)$ and $\phantom {\dot {i}\!}\Pi _{\rho, \mathbf {c}_{n}} \cdot $ denotes the patch extraction operator with center **c**_*n*_ and radius *ρ*. In order to perform a multi-object segmentation, we consider the following multi-ellipse optimization problem:

18 $$\begin{array}{*{20}l}  & \underset{{{{\Gamma}}}_{1}, \hdots, {{{\Gamma}}}_{N}}{\arg\min} \ \sum_{n=1}^{N}\! \left\lbrace\! \frac{1}{a_{n}b_{n}}\! \sum_{\mathbf{x}}\! w_{\epsilon} \! \left(\! \|\mathbf{x} - {\mathbf{x}_0}^{n} \|_{_{{{\Gamma}}_{n}}}^{2} \! \right) \! \Pi_{\rho, \mathbf{c}_{n}} u\left[\mathbf{x}\right] \right\rbrace \\ & \text{subject to} \left({{{\Gamma}}}_{1}, {{{\Gamma}}}_{2}, \hdots, {{{\Gamma}}}_{N} \right) \in \Upsilon \,  \end{array} $$

where $\left \lbrace {\mathbf {x}_{0,n}}, a_{n}, b_{n}, \theta _{n} \right \rbrace $ are the parameters of the ellipse *Γ*_*n*_ and *Υ* is a set of constraints to ensure the ellipses fall into an acceptable range of configurations. In practice, we typically set

$$\begin{array}{*{20}l} \!\!\!\!\!\!\!\!\!\!\!\! \Upsilon & = \big\lbrace \left\| {\mathbf{x}_{0,n}} - {\mathbf{x}_{0,n'}} \right\|_{2} > \rho;~~~~ \\ & \quad \quad \quad \quad \ \left\| {\mathbf{x}_{0,n}} - \mathbf{c}_{n} \right\|_{\infty} \leq \rho_{\max};~~~~ \\ & \quad \quad \quad \quad \ r_{\min} \leq a_{n}, b_{n} \leq r_{\max};~~~~ \\ & \quad \quad \quad \quad \ \theta_{\min} \leq \theta_{n} \leq \theta_{\max} \quad \quad \quad \big\rbrace_{1 \leq n, n'\leq N} \, \end{array} $$

for some predefined values $\rho _{\max }, r_{\min }, r_{\max }, \theta _{\min }, \theta _{\max }$ set according to the extracted patch positions and the allowed sizes and orientations of tissue cores. The constraint $\left \| {\mathbf {x}_{0,n}} - {\mathbf {x}_{0,n'}} \right \|_{2} > \rho $ which prevents the distance between two ellipse centers being too close helps to avoid the overlapping of segmented tissue cores. In what follows, we denote $\mathcal {J}(u, \Gamma _{1}, \ldots, \Gamma _{n})$ the global cost function associated with the optimization problem (18).

By construction, the function $\mathcal {J}(u, \Gamma _{1}, \ldots, \Gamma _{n})$ is differentiable with respect to $\left ({{\Gamma }}_{1}, \hdots, {{\Gamma }}_{N} \right)$. The common way to minimize $\mathcal {J}(u, \Gamma _{1}, \ldots, \Gamma _{n})$ under the constraint set *Υ* is to use a gradient method whose performance depends on how efficient is the computation of the gradient of $\mathcal {J}(u, \Gamma _{1}, \ldots, \Gamma _{n})$. Since $\mathcal {J}(u, \Gamma _{1}, \ldots, \Gamma _{n})$ is a linear combination of separable functions, therefore, the gradient can be simply obtained as:

19 $$ \nabla \mathcal{J}(u, \Gamma_{1}, \ldots, \Gamma_{n}) = \left(\begin{array}{c} \nabla J (\Pi_{\rho,\mathbf{c}_{1}} u, \Gamma_{1}) \\ \vdots \\ \nabla J (\Pi_{\rho,\mathbf{c}_{N}} u, \Gamma_{N}) \end{array} \right),  $$

where

$$J (\Pi_{\rho,\mathbf{c}_{n}} u, \Gamma_{n}) = \frac{1}{a_{n}b_{n}} \sum_{\mathbf{x} \in \Gamma_{n}}\! w_{\epsilon} \left(\|\mathbf{x} - {\mathbf{x}_0}^{n} \|_{_{{{\Gamma}}_{n}}}^{2} \right) \Pi_{\rho,\mathbf{c}_{n}} {u}[\mathbf{x}] $$

and the expressions of its partial derivatives are given in (17).

The result of the multi-ellipse optimization problem (18) is a set of ellipses $\left \lbrace {{\Gamma }}_{n} \right \rbrace _{1 \leq n \leq N}\phantom {\dot {i}\!}$ which fits the objects located in the regions of interest $\lbrace \Pi _{\rho,\mathbf {c}_{n}} u \rbrace _{1 \leq n \leq N}\phantom {\dot {i}\!}$. Furthermore, given the major axes of these ellipses and the TMA core radius *r*_*core*_, we discard the tiny, giant and flattened ellipses and we keep those which are most similar to the expected tissue cores. The center of the selected ellipse allows us to determine the position of the recognized TMA core. This reference position will be used to determine the array coordinates of the corresponding tissue core. In the following, we denote $\mathcal {X}_{0} = \lbrace {\mathbf {x}_{0,n}} \rbrace _{n \in \lbrace 1, \hdots, N \rbrace }$ as the set of centers of the *N* reliable and selected ellipses.

### Estimation of array coordinate and TMA core positions

**Fig. 9:**
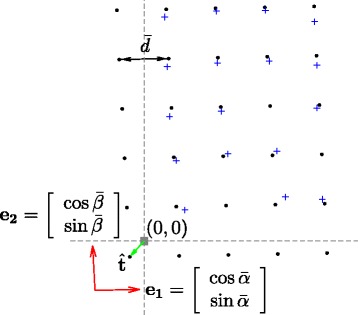
Affine approximation of the grid deformation. The distorted grid *Λ* which one only observes partially the set of point $\mathcal {X}_{0} \subset \Lambda $ (shown in blue crosses) is approximated by the oblique (regular) grid *Λ*_0_ (black circled dots). The latter is characterized by the average distance $\bar {d}$ between its points, two principal directions which are presented by two vectors (*e*_1_,*e*_2_) (red arrows), and the global translation $\hat {\mathbf {t}}$ (green arrow) of the grid with respect to the origin (0,0) (gray square dot)

An ideal TMA is the one which has tissue cores perfectly aligned in both horizontal and vertical directions and equally spaced according to a regular square grid. The array coordinate $\mathbf {p} = (k, l) \in \mathbb {Z}^{2}$ of a core can be simply obtained by drawing two orthogonal lines crossed at the considered core position. However, due to the deformation of the design TMA grid, the lines passing through tissue cores and their nearest neighbors may be slightly inclined with respect to the horizontal or vertical axes. Moreover, the direction of these lines may have a large spectrum of variations which makes more challenging the tracking of tissue cores over a given direction. To deal with this deformation, existing TMA de-arraying methods use usually distance-and-angle-based criteria for the purpose of defining the neighborhood of TMA cores. Although this approach estimates robustly the average core-to-core distance and the two principal directions of the deformed core grid, it may fail for some well-detected cores whose the position is strongly distorted with respect to their neighbors. In order to avoid this failure, we introduce an algorithm for estimating iteratively the deformation of the TMA grid in a way that the grid which is warped by the estimated deformation at an iteration gets closer to the observed TMA grid. To this end, we assume that the deformation of the TMA grid can be decomposed by linear and non-linear parts. Under this assumption, we estimate the linear part of the deformation by defining an oblique grid (affine warping) which is derived from the detected core positions as the initialization of the warped grid (see Fig. 9). The latter is used to find nearby cores that will be taken into account to compute an estimator of the grid deformation by using the thin-plate interpolation [25] if we do an analogy with material deformation.

### Estimation of the linear deformation

Our goal is to approximate the distorted TMA grid *Λ* (which is observed partially with the set of point $\mathcal {X}_{0}$) by an oblique grid *Λ*_0_ which minimizes the distance between them in the way that the deformation of the grid is approximated by a 2D affine transform. For this purpose, we consider the set ${\mathcal {C}_0}$ of core pairs whose each pair $\phantom {\dot {i}\!}({\mathbf {x}_{0,n}}, {\mathbf {x}_{0,n'}})$ is formed by an element of $\mathcal {X}_{0}$ and one of its four nearest neighbors with respect to the Euclidean distance

$$ {\mathcal{C}_0} = \left\lbrace ({\mathbf{x}_{0,n}}, {\mathbf{x}_{0,n'}}) \in \mathcal{X}_{0} \times \mathcal{X}_{0},\, {\mathbf{x}_{0,n'}} \in \mathcal{N}({\mathbf{x}_{0,n}}) \right\rbrace, $$

where $\mathcal {N}({\mathbf {x}_{0,n}})$ denotes the 4-neighborhood of **x**_0,*n*_. To estimate the average core-to-core distance $\bar {d}_{cc}$, we compute the trimmed mean (denoted TM) of the length of the segment defined by the pair $\phantom {\dot {i}\!}({\mathbf {x}_{0,n}}, {\mathbf {x}_{0,n'}})$ of ${\mathcal {C}_0}$ by discarding the most extreme values (typically 30%):

20 $$ {\bar{d}} = \text{TM}_{30\%} \left\lbrace \| {\mathbf{x}_{0,n}} - {\mathbf{x}_{0,n'}}\|_{2} \right\rbrace_{({\mathbf{x}_{0,n}}, {\mathbf{x}_{0,n'}}) \in {\mathcal{C}_0}} \ .  $$

Let $\text {ang}({\mathbf {x}_{0,n}}, {\mathbf {x}_{0,n'}})\phantom {\dot {i}\!}$ be the angle between the line passing through $({\mathbf {x}_{0,n}}, {\mathbf {x}_{0,n'}})\phantom {\dot {i}\!}$ and the horizontal axis such that $-0.25{\pi } \leq \text {ang}({\mathbf {x}_{0,n}}, {\mathbf {x}_{0,n'}}) \leq {0.75\pi }\phantom {\dot {i}\!}$. By analogy, we define the two principal angles of the deformed TMA grid as follows:

$$\begin{array}{*{20}l} {\bar{\alpha}} & = \text{TM}_{30\%} \left\lbrace \text{ang}({\mathbf{x}_{0,n}}, {\mathbf{x}_{0,n'}}) \leq \frac{\pi}{4} \right\rbrace, \\ {\bar{\beta}} & = \text{TM}_{30\%} \left\lbrace \text{ang}({\mathbf{x}_{0,n}}, {\mathbf{x}_{0,n'}}) \geq \frac{\pi}{4} \right\rbrace. \end{array} $$

Finally, we denote $\hat {\mathbf {t}}$ as the global translation of the distorted TMA grid with respect to the origin that minimizes the distance between the set $\mathcal {X}_{0}$ and the linearly-estimated grid *Λ*_0_

21 $$ \hat{\mathbf{t}} = \arg\min_{\mathbf{t}} \sum_{n = 1}^{N} \min_{\mathbf{p} \in \mathbb{Z}^{2}} \| \mathbf{t} + \mathcal{F}(\mathbf{p}) - {\mathbf{x}_{0,n}}\|_{2}^{2}  $$

where $\mathcal {F}$ maps each array coordinates $\mathbf {p} \in \mathbb {Z}^{2}$ to a position of *Λ*_0_ corrected by $\hat {\mathbf {t}}$ and

22 $$ \mathcal{F}(\mathbf{p}) = \bar{d} \ \underset{\mathbf{M}_{\bar{\alpha}, \bar{\beta}}}{\underbrace{\left[ \!\! \begin{array}{c c} \cos\bar{\alpha} & \cos\bar{\beta} \\ \sin\bar{\alpha} & \sin\bar{\beta} \end{array} \!\! \right] }} \ \mathbf{p} \ .  $$

Note that $\mathbf {M}_{\bar {\alpha }, \bar {\beta }}$ is a change-of-basis matrix of unit column vectors and $\bar {d}$ is a scaling factor which transforms array coordinates (elements of $\mathbb {Z}^{2}$) to real spatial positions (in $\Omega \subset \mathbb {R}^{2}$). The resulting oblique grid is parametrized with four parameters $\{\bar {d}, \bar {\alpha }, \bar {\beta }, \hat {t}\}$ and represents the affine part of the grid deformation. We thus arrive at the affine mapping function: $\mathcal {A}(\mathbf {p}) = \hat {\mathbf {t}} + \mathcal {F}(\mathbf {p}) \in \Lambda _{0}$. The oblique grid *Λ*_0_ will serve as initialization to estimate of the non-linear deformation of the grid. Figure 9 illustrates an example showing the oblique grid obtained from a given set of points as well as its estimated parameters.

### Thin-plate-based estimation of the deformation

Let *Λ*^⋆^ be the ideal design TMA grid with (0,0) as origin and *d* the ideal distance between two neighboring cores along the horizontal and vertical axes. The mapping is then defined as:

$$ {\mathbf{y}_{\mathbf{p}}^{\star}} = d \mathbf{p} \in {{\Lambda^{\star}}}, \forall \mathbf{p} \in \mathbb{Z}^{2}. $$

The deformation ${\mathcal {D}}$ maps each point ${\mathbf {y}_{\mathbf {p}}^{\star }} \in {{\Lambda ^{\star }}}$ onto a point ${\mathbf {y}_{\mathbf {p}}} = \mathcal {D}({\mathbf {y}_{\mathbf {p}}^{\star }})$ in the distorted grid *Λ*. In order to estimate the deformation ${\mathcal {D}}$ at all points of the grid *Λ*^⋆^, we aim at approximating this set from the observed set $\mathcal {X}_{0} = \{{\mathbf {x}_{0,n}}\} $ by using the thin-plate splines as an interpolant. Indeed, given a set of points ${\mathcal {D}}^{-1} \mathcal {X}_{0} = \left \lbrace {\mathcal {D}}^{-1} \left ({\mathbf {x}_{0,n}} \right) \right \rbrace _{n \in \lbrace 1, \ldots, N \rbrace }$, the coefficients of the interpolating thin-plate splines are the minimizers of a quadratic function which is the first approximation of the bending energy of the mapping from ${\mathcal {D}}^{-1} \mathcal {X}_{0}$ to the set of target points $\mathcal {X}_{0}$ (see [25]). Nevertheless, unlike the usual framework [25], the correspondence between the two sets of points is not established, that is ${\mathcal {D}}^{-1} \mathcal {X}_{0}$ is unknown. Instead of investigating a matching method to determine ${\mathcal {D}}^{-1} \mathcal {X}_{0}$, we propose to build a sequence of grids {*Λ*^(*m*)^}_*m*≥0_ which evolves iteratively to fit $\mathcal {X}_{0}$. We initialize this sequence with the oblique grid *Λ*^(0)^=*Λ*_0_ previously computed. The linear approximation of ${\mathcal {D}}$ is then as follows:

23 $$\begin{array}{@{}rcl@{}} {\mathbf{y}_{\mathbf{p}}}^{(0)}&=& {\mathcal{D}}^{(0)}({\mathbf{y}_{\mathbf{p}}^{\star}}) \\ &=& \hat{\mathbf{t}} + \frac{\ \bar{d}\ }{\ {d} \ }\!\left[ \!\! \begin{array}{c c} \cos\bar{\alpha} & \cos\bar{\beta} \\ \sin\bar{\alpha} & \sin\bar{\beta} \end{array} \!\! \right]\! \mathbf{p}.  \end{array} $$

**Fig. 10:**
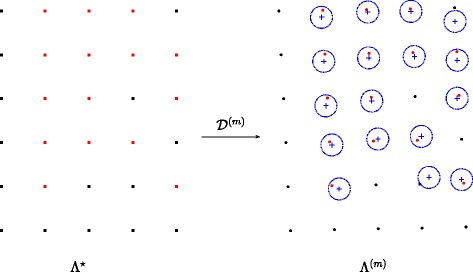
Correspondence between the ideal grid and the observed distorted grid. At iteration *m*, the estimated deformation $\mathcal {D}^{(m)}$ maps each point $\mathbf {y}^{\star }_{\mathbf {p}}$ of the ideal square grid *Λ*^⋆^ (shown in square dots on the left) onto a point $\mathbf {y}^{(m)}_{\mathbf {p}} = \mathcal {D}^{(m)} \big (\mathbf {y}^{(m)}_{\mathbf {p}}\big) $ in the warped grid *Λ*^(*m*)^ (circled dots on the right) which fits the observed set of points $\mathcal {X}_{0}$ (blue crosses). A position $\mathbf {x}_{0, n} \in \mathcal {X}_{0}$ is associated to a position $\mathbf {y}^{\star }_{\mathbf {p}}$ if **x**_0,*n*_ is located within a radius *δ* from $\mathbf {y}^{(m)}_{\mathbf {p}}$ (blue dotted circles). Associated positions are marked in red

At iteration *m*, a core position ${\mathbf {x}_{0,n}} \in \mathcal {X}_{0}$ is associated to a position ${\mathbf {y}_{\mathbf {p}}^{\star }}$ if the former is located within a radius *δ* from ${\mathbf {y}_{\mathbf {p}}}^{{{(m)}}} = {\mathcal {D}}^{{{(m)}}}({\mathbf {y}_{\mathbf {p}}^{\star }})$. Pairs of associated positions establish therefore the correspondence between the ideal grid *Λ*^⋆^ and the set of observed point $\mathcal {X}_{0}$. We also note that all the positions of *Λ*^⋆^ do not have a corresponding position in $\mathcal {X}_{0}$ as shown in Fig. 10 mainly because the cardinality of these sets are not the same. Let $\mathcal {P}^{{{(m)}}}$ be the set of pairs of associated positions:

24 $$ \mathcal{P}^{{{(m)}}} = \left\lbrace \left({\mathbf{x}_{0,n}}, {\mathbf{y}_{\mathbf{p}}^{\star}} \right),\, \| {\mathcal{D}}^{{{(m)}}}({\mathbf{y}_{\mathbf{p}}^{\star}}) - {\mathbf{x}_{0,n}} \|_{2} \leq \delta \right\rbrace.~~~~  $$

Assume that *N*^(*m*)^ is the number of elements of $\mathcal {P}^{{{(m)}}}$. The objective is to estimate the deformation $\mathcal {D}^{{{(m)}}}$ from the set of *N*^(*m*)^ associated pairs $({\mathbf {x}_{0,n}}, {\mathbf {y}_{\mathbf {p}}^{\star }})$. According to [25], we define the Gram’s matrix $\left \lbrace \mathbf {K}^{{{(m)}}}_{n, n'}\right \rbrace _{1 \leq n, n' \leq N^{{{(m)}}}}$ as follows:

25 $$\begin{array}{@{}rcl@{}} \mathbf{K}^{{{(m)}}}_{n, n'} & = \left\| {\mathbf{x}_{0,n}} - {\mathbf{x}_{0,n'}} \right\|_{2}^{2} \log \left\| {\mathbf{x}_{0,n}} - {\mathbf{x}_{0,n'}} \right\|_{2}^{2}, \end{array} $$

and the additional matrices as:

26 $$\begin{array}{@{}rcl@{}} \mathbf{Y}^{{{(m)}}} & = &\left[ \begin{array}{cccc} 1 & 1 & \hdots & 1 \\ \mathbf{y}_{\mathbf{p}_{1}} & \mathbf{y}_{\mathbf{p}_{2}} & \hdots & \mathbf{y}_{\mathbf{p}_{N^{(m)}}} \end{array} \right], \end{array} $$

27

28 $$\begin{array}{@{}rcl@{}} \mathbf{X}^{{{(m)}}} & = &\left[ \begin{array}{ccccccc} \mathbf{x}_{0,1} & \mathbf{x}_{0,2} & \hdots & \mathbf{x}_{0,N^{{{(m)}}}} & 0 & 0 & 0\\ \end{array} \right], \end{array} $$

29 $$\begin{array}{@{}rcl@{}} \mathbf{W}^{{{(m)}}} & = & \left(\left(\mathbf{L}^{{{(m)}}}\right)^{-1} \left(\mathbf{X}^{{{(m)}}}\right)^{\top}\right)^{\top}, \end{array} $$

and $\mathbf {W}^{{{(m)}}} \,=\, \left (\!\mathbf {w}_{1}^{{{(m)}}}, \mathbf {w}_{2}^{{{(m)}}}, \hdots, \mathbf {w}_{N^{{{(m)}}}}^{{{(m)}}}\!\right)^{\top }$. By using the entries of the matrix **W**^(*m*)^, the estimators of the deformation ${\mathcal {D}}$ and of the grid *Λ* at the next iteration *m*+1 are therefore defined as:

30 $$\begin{array}{@{}rcl@{}} {\mathbf{y}_{\mathbf{p}}}^{(m + 1)} & = & \mathbf{w}^{^{{{(m)}}}}_{_{N^{{{(m)}}} + 1}} + \left[\mathbf{w}^{^{{{(m)}}}}_{_{N^{{{(m)}}} + 2}} \mathbf{w}^{^{{{(m)}}}}_{_{N^{{{(m)}}} + 3}} \right] {{\mathbf{y}_{\mathbf{p}}^{\star}}}\\  && + \sum_{n=1}^{N^{{{(m)}}}} \mathbf{w}^{{{(m)}}}_{n} \left(\| {\mathbf{x}_{0,n}} - {{\mathbf{y}_{\mathbf{p}}^{\star}}}\|_{2}^{2} \log \| {\mathbf{x}_{0,n}} - {{\mathbf{y}_{\mathbf{p}}^{\star}}}\|_{2}^{2}\right). \end{array} $$

This iterative scheme will be stopped at the iteration *m*^∗^=*m* if there are no change between *Λ*^(*m*)^ and *Λ*^(*m*+1)^. At convergence, the row and column coordinates of a detected cores of position ${\mathbf {x}_{0,n}} \in \mathcal {X}_{0}$ is simply given by:

31 $$ \hat{\mathbf{p}} = \underset{\mathbf{p} \in \mathbb{Z}^{2}}{\arg\min} \big\| {\mathbf{x}_{0,n}} - \mathcal{D}^{(m^{*})}({\mathbf{y}_{\mathbf{p}}^{\star}})\big\|_{2}^{2} \ .  $$

Moreover, since the grid ${{\Lambda }}^{(m^{*})}$ is an estimator of the deformed TMA grid *Λ* which is partially observed in $\mathcal {X}_{0}$, it can be used as approximated positions to recognize tissue cores which are missed during detection and segmentation processes. Indeed, to refine de-arraying result, we perform another multi-ellipse optimization at the position of remaining nodes of the grid ${{\Lambda }}^{(m^{*})}$. If there are ellipses that meet the size and the roundness criteria of standard cores, we add them to the list of detected core position and adjust the coefficients of the thin-plate splines according to the new list. An example of TMA de-arraying is depicted in Fig. 14 showing the gain of our method in term of tissue core detection.

## Results and discussion

### Description of data sets

To evaluate our de-arraying ATMAD approach, we selected a number of DNA microarray and tissue microarray images including those which are artificially simulated and those which are acquired in both brightfield and fluorescence modes. The selected images were collected from various sources and can be classified into three data sets.

The first set is a collection of binary images generated by Dr Yinhai Wang in [22] as pseudo TMA slides. This data set was artificially created by taking account of different possible situations occurring during the TMA manufacturing process, including rotations and stretches of the design grid as well as irregularities in the size and the shape of tissue cores. The average core radius is approximately *r*_*core*_=15 pixels for all images. The whole set of all these simulated images and ground truths can be freely downloaded at https://get.google.com/albumarchive/117531880452844036890.

The second data set is composed of color TMA images from the AIDS and Cancer Specimen Resource (ACSR) Digital Library of the University of California San Francisco (http://acsr.ucsf.edu). This online library – managed and visualized by Aperio’s WebScope software – contains several hundreds of tissue specimens which are mostly stained with H&E (Hematoxylin and Eosin) stain and are imaged by brightfield microscopy technique. For this experiment, we considered down-sampled version (with the magnification between 0.4X and 0.6X) of the original images hosted on ACSR’s server in order to reduce the processing time. The considered resolutions correspond to images of approximately 1000×1000 pixels, on which the TMA cores have radius of only a few dozen pixels but it is sufficient for our approach to localize them.

The third set for the evaluation includes fluorescence high-dynamic-range (HDR) images showing DNA microarray and tissue microarray slides. Provided by the courtesy of Innopsys company, these HDR images which were saved in 16-bit-TIFF format were acquired using a scanner called InnoScan 1100AL (see https://www.innopsys.com/en/lifesciences-products/microarrays/innoscan/innoscan-1100-al for more details). The latter which is equipped with three excitation lasers (488 nm, 532 nm and 625 nm compatible with cyanine dyes such as Cy2, Cy3 and Cy5 respectively). It can perform simultaneously the acquisition on each excitation channel and provides up to three color fluorescence images. The maximal scan area supported by the mentioned device is 22×74 mm^2^ corresponding to the size of typical microscopy slides used in most biological laboratories nowadays. For the same reason with ACSR’s images, we selected typical images acquired by this Innopsys’s scanner with spatial resolutions varying in a range from 10 to 40 *μ*m per pixel in this experiment instead of using those with higher resolution (up to 0.5 *μ*m per pixel or a 20X magnification equivalently). Indeed, considering such images of low resolution and small size as input data not only enables efficient and low-memory-requirement processing but also requires very short scanning time – less than just five minutes with a resolution of 10 *μ*m per pixel when compared with typically several hours of acquisition at sub-micrometer resolutions.

**Fig. 11:**
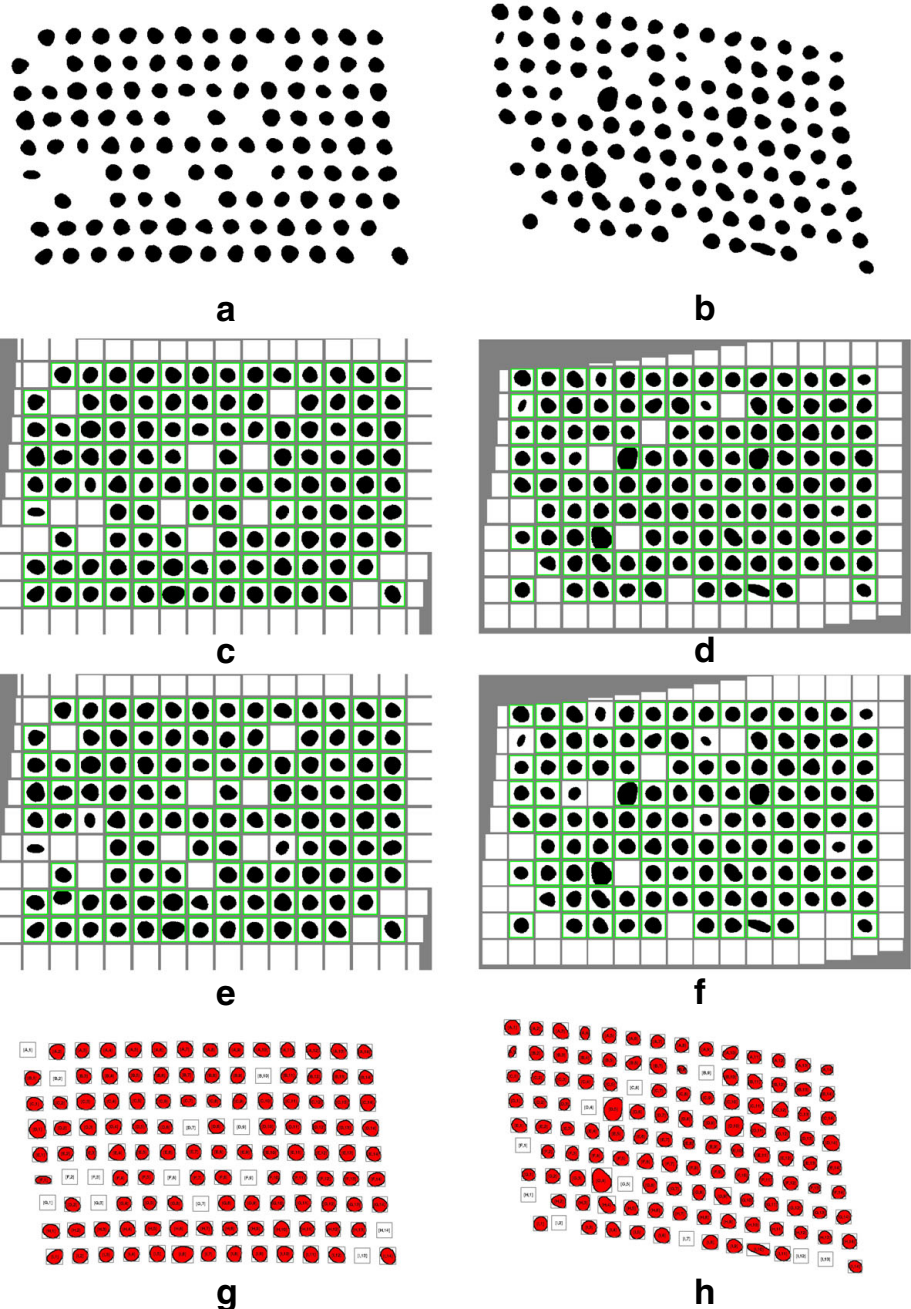
Example of de-arraying on simulated images. From left to right : TMAs with the grid deformation varying from low to high. From top to bottom : original images, de-arraying result by the proposed method with segmentation module deactivated/activated, ground truth given by Dr Jinhai Wang. The obtained de-arraying results are presented in array form with recognized spot positions marked by green boxes

**Fig. 12:**
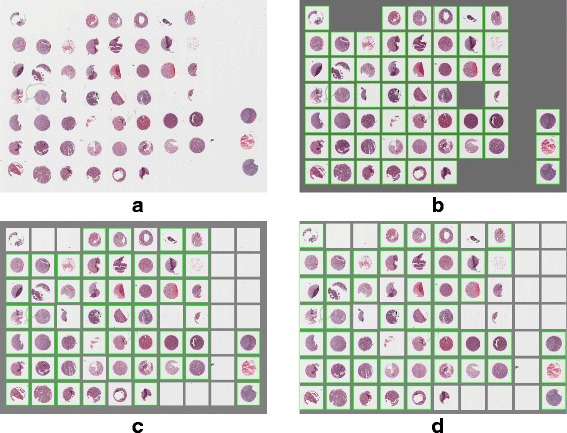
Example of de-arraying on brightfield TMA image. **a** Original image : H&E stained TMA on ACSR’s database with ID 550-T0011-01. **b** Manual annotation used for comparison with de-arraying results. **c**-**d** De-arraying results obtained with the deactivation/activation of the segmentation module. These results and the manual annotation are represented in array format with recognized cores marked by green boxes

**Fig. 13:**
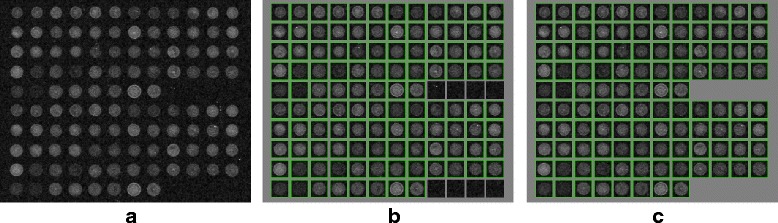
Example of de-arraying on a fluorescence DNA microarray image with the deactivation of both the segmentation and of the non-linear estimation for the TMA grid deformation. **a** Contrast-enhanced original image. **b** De-arraying result of the proposed method presented in array format. **c** Manual annotations in array format. For comparison purpose, recognized DNA spots are marked by green boxes

**Fig. 14:**
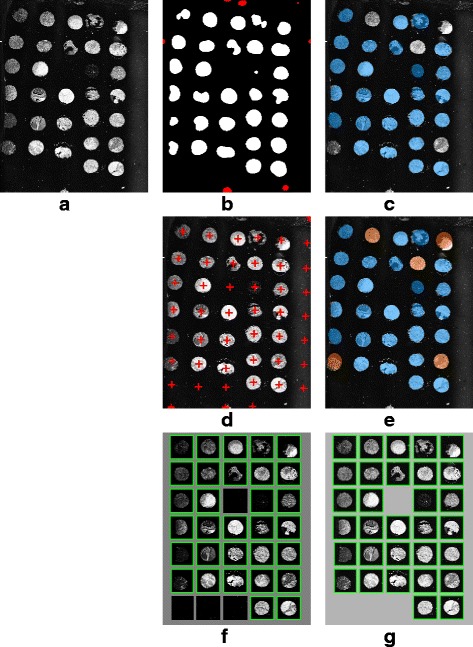
Example of de-arraying on a fluorescence TMA image with the activation of both the segmentation and of the non-linear estimation for the TMA grid deformation. **a** Contrast-enhanced original image. **b** Detection map (accurate detection is marked in white, wrong detection is marked in red). **c** Segmentation of TMA cores (recognized cores are colored by blue ellipses). **d** Estimated TMA grid (potential core position is marked by a red cross). **e** Recognized TMA cores (cores which are additionally recognized are colored by orange ellipses). **f** Final de-arraying result in array format (recognized core position is marked by green box). **g** Manual annotations for comparison

Regarding the complexity of the data sets, it contains difficult cases such as irregular and non-rounded shapes, fragmented cores as well as low contrasts between image background and foreground. Sophisticated array design with incomplete (missing cores) rows and columns is also present in the image set for the purpose of testing the robustness of our de-arraying approach (see Figs. 11, 12, 13 and 14).

### Experimental results and algorithm evaluation

For the first and second data sets which contain images with dark spots and bright background, we firts performed a color inversion before further processing. The de-arraying procedure was directly applied on binary and grayscale images. Multi-channel color images as in the case of ACSR’s data require a conversion to grayscale such as a simple average over all channels which we used in these experiments.

In order to evaluate the performance of our ATMAD algorithm, we analyzed the obtained results by considering two criteria: (i) the rate of samples which are successfully localized and (ii) the rate of samples whose array coordinates are correctly estimated. To that end, the de-arraying ATMAD outcome was compared with the ground-truth provided by the simulated dataset or by manual annotation of real-world TMA images. The comparative similarity between the de-arraying results and ground-truths (simulation, annotation) is quantitatively measured by these six following metrics:

- Accuracy: $\mathrm {A} = \frac {\text {TP} + \text {TN}}{\text {TP} + \text {TN} + \text {FP} + \text {FN}}$,
- Precision: $\mathrm {P} = \frac {\text {TP}}{\text {TP} + \text {FP}}$,
- Recall (sensitivity): $\mathrm {R} = \frac {\text {TP}}{\text {TP} + \text {FN}}$,
- F-score: $\mathrm {F} = 2\frac {\mathrm {P}\mathrm {R}}{\mathrm {P} + \mathrm {R}}$,
- G-score: $\mathrm {G} = \sqrt {\mathrm {P} \mathrm {R}}$,
- Jaccard coefficient: $\text {JSC} = \frac {\text {TP}}{\text {TP} + \text {FP} + \text {FN}}$.

“True Positive” (TP) denotes the number of true tissue samples (cores) which are correctly localized, or those whose array coordinates are correctly estimated. “False Negative” (FN) denotes the number of true cores which are not successfully localized (due to non detection or failed segmentation), or those whose array coordinates are not estimated. “False Positive” (FP) denotes the number of cores which are wrongly localized (due to false detection), or those whose array coordinates are wrongly estimated. “True Negative” (TN) denotes the number of “empty” spot positions (no core is placed) where no core is wrongly localized.

To better appreciate the impact of the components (or modules) of our de-arraying approach, the performance was evaluated under four different setting options (see also Table 1):

**Table 1:** Workflow options corresponding to the selection (activation/deactivation) of ellipse-based segmentation and non-linear registration modules

	Ellipse-based	Non-linear
	segmentation	registration
Option #1	–	–
Option #2	$\bigstar $	–
Option #3	–	$\bigstar $
Option #4	$\bigstar $	$\bigstar $

1. deactivation of ellipse-based segmenta-
  tion and non-linear registration modules,
2. activation of ellipse-based segmentation
  module and deactivation of non-linear
  registration module,
3. deactivation of ellipse-based segmenta-
  tion module and activation of on-linear
  registration module,
4. activation of ellipse-based segmentation
  and non-linear registratione modules.

When the ellipse-based segmentation module is deactivated, the spot localization is performed only with the wavelet-based detection method. Consequently, the process of removal of unreliable detected cores based on the size and the shape criteria is then disable, and the refinement of de-arraying result using estimated positions of the deformed TMA grid can not be performed. Meanwhile, the deactivation of the non-linear registration module implies that the grid deformation is assumed to be approximated by an affine (linear) transform. It could result in a non-coincidence between core positions and estimated positions of the deformed grid for most of cores. A distance-based matching is thus necessary to establish the correspondence of each core position and its array coordinates. To allow a step-by-step evaluation of the performance, besides the final de-arraying result, intermediary results of the de-arraying procedure were also carefully analyzed.

For a comparative evaluation, we also provide de-arraying results on simulated images (which are generated using the deformation model described in [22]) obtained with the Wang’s method [22] – the state-of-the-art method for TMA brightfield image de-arraying – and compare these results to those obtained with the proposed ATMAD method. Unfortunately, it was not possible to apply the method [22] on real-world images since the software and code are not available. In Table 2, the average performance obtained on each dataset as well as on each example is shown in Figs. 11, 12, 13 and 14. We notice that, except the precision and recall scores which are not in agreement in certain cases, the Accuracy, F-score, G-score and Jaccard metrics yield consistent results about the effectiveness of the de-arraying method. Accordingly, we will focus on the F-score metric in the next sections for the sake of simplicity. The results with all the metrics are reported in Table 2.

**Table 2:** Performance of the proposed de-arraying method on three datasets under four setting options: (1) both the segmentation and the non-linear estimation (for the deformation) modules are deactivated, (2) the segmentation is activated but the non-linear estimation is deactivated, (3) the segmentation is deactivated and the non-linear estimation is activated, and (4) both of them are activated

	Localization						Estimation of array coordinates					
	A ^(a)^	P ^(b)^	R ^(c)^	F ^(d)^	G ^(e)^	JSC ^(f)^	A ^(a)^	P ^(b)^	R ^(c)^	F ^(d)^	G ^(e)^	JSC ^(f)^
SIMULATED IMAGES												
*Average on 31 images*												
Wang et al. [22]	1.00	1.00	1.00	1.00	1.00	1.00	1.00	1.00	1.00	1.00	1.00	1.00
ATMAD Opt. #1	1	1	1	1	1	1	0.93	1	0.93	0.96	0.96	0.93
Opt. #2	0.95	1	0.95	0.98	0.98	0.95	0.85	1	0.84	0.91	0.91	0.84
Opt. #3	1	1	1	1	1	1	1	1	1	1	1	1
Opt. #4	0.95	1	0.95	0.98	0.98	0.95	0.95	1	0.95	0.98	0.98	0.95
*Fig. 11a*												
ATMAD Opt. #1	1	1	1	1	1	1	1	1	1	1	1	1
Opt. #2	0.97	1	0.96	0.98	0.98	0.96	0.97	1	0.96	0.98	0.98	0.96
Opt. #3	1	1	1	1	1	1	1	1	1	1	1	1
Opt. #4	0.97	1	0.96	0.98	0.98	0.96	0.97	1	0.96	0.98	0.98	0.96
*Fig. 11b*												
ATMAD Opt. #1	1	1	1	1	1	1	0.93	1	0.92	0.96	0.96	0.92
Opt. #2	0.93	1	0.92	0.96	0.96	0.92	0.90	1	0.89	0.94	0.95	0.89
Opt. #3	1	1	1	1	1	1	1	1	1	1	1	1
Opt. #4	0.93	1	0.92	0.96	0.96	0.92	0.93	1	0.92	0.96	0.96	0.92
BRIGHTFIELD IMAGES												
*Average on 8 images*												
ATMAD Opt. #1	0.94	1	0.93	0.96	0.96	0.93	0.91	1	0.88	0.94	0.94	0.88
Opt. #2	0.87	1	0.83	0.91	0.91	0.83	0.84	1	0.79	0.88	0.89	0.79
Opt. #3	0.94	1	0.93	0.96	0.96	0.93	0.94	1	0.93	0.96	0.96	0.93
Opt. #4	0.87	1	0.83	0.91	0.91	0.83	0.93	1	0.91	0.95	0.95	0.91
*Fig. 12*												
ATMAD Opt. #1	0.96	1	0.94	0.97	0.97	0.94	0.91	1	0.89	0.94	0.94	0.89
Opt. #2	0.87	1	0.83	0.91	0.91	0.83	0.84	1	0.80	0.89	0.89	0.80
Opt. #3	0.96	1	0.94	0.97	0.97	0.94	0.96	1	0.94	0.97	0.97	0.94
Opt. #4	0.87	1	0.83	0.91	0.91	0.83	0.93	1	0.91	0.95	0.95	0.91
FLUORESCENCE IMAGES												
*Average on 8 DNA microarray images*												
ATMAD Opt. #1	1	1	1	1	1	1	1	1	1	1	1	1
Opt. #2	1	1	1	1	1	1	1	1	1	1	1	1
Opt. #3	1	1	1	1	1	1	1	1	1	1	1	1
Opt. #4	1	1	1	1	1	1	1	1	1	1	1	1
*Fig. 13*												
ATMAD Opt. #1	1	1	1	1	1	1	1	1	1	1	1	1
Opt. #2	1	1	1	1	1	1	1	1	1	1	1	1
Opt. #3	1	1	1	1	1	1	1	1	1	1	1	1
Opt. #4	1	1	1	1	1	1	1	1	1	1	1	1
*Average on 4 TMA images*												
ATMAD Opt. #1	0.79	0.77	1	0.87	0.88	0.77	0.92	0.98	0.93	0.95	0.95	0.91
Opt. #2	0.91	0.99	0.90	0.94	0.95	0.89	0.73	1	0.69	0.82	0.83	0.69
Opt. #3	0.79	0.77	1	0.87	0.88	0.77	-	-	-	-	-	-
Opt. #4	0.91	0.99	0.90	0.94	0.95	0.89	0.92	0.98	0.93	0.96	0.96	0.91
*Fig. 14*												
ATMAD Opt. #1	0.88	0.86	1	0.93	0.93	0.86	0.94	1	0.94	0.97	0.97	0.94
Opt. #2	0.86	1	0.84	0.91	0.92	0.84	0.80	1	0.77	0.87	0.88	0.77
Opt. #3	0.88	0.86	1	0.93	0.93	0.86	0.97	1	0.97	0.98	0.98	0.97
Opt. #4	0.86	1	0.84	0.91	0.92	0.84	1	1	1	1	1	1

All considered performance scores range from 0 (worst) to 1 (best) and measure the similarity between the de-arraying results and their corresponding ground-truth or manual annotation. Notations: ^(a)^ accuracy (A), ^(b)^ precision (P), ^(c)^ recall (R), ^(d)^ F-score (F), ^(e)^ G-score (G) and ^(f)^ Jaccard coefficient (JSC)

### Simulated images

We evaluated our ATMAD method applied to the Wang’s dataset and we compared the results with those obtained with the method described in [22].

An example of de-arraying result with different levels of deformation is illustrated in Fig. 11. The top row shows the original images. The two middle rows show the de-arraying outcomes obtained with deactivation and activation of the segmentation respectively (the non-linear estimation for the deformation is activated in both cases). These two cases correspond to the Option #3 and #4 respectively, as reported in Table 2. The recognized spot positions are marked by green boxes and correctly aligned in a array to facilitate localization and identification. The bottom row of Fig. 11 shows the ground-truth provided by the authors of the dataset.

As expected, in the case of simulated images when the background is constant, our method provided a perfect F-score = 1 (corresponding to an accuracy of 100%) in average with the Option #3 even if the localization of spots is only performed with the wavelet-based detection method. On the second row of Fig. 11 showing the de-arraying results obtained on two typical examples with the deactivation of the ellipse-based segmentation method, we notice that all the spots are successfully recognized and the array coordinates are correctly estimated. The results are similar to those obtained with the Wang’s method [22] (for more details, see Table 2). Meanwhile, the de-arraying results obtained with the Option #4 achieved a slightly lower F-score F=0.98 (corresponding to an accuracy of 95%) in average. This score is a direct consequence of the fact that all existing spots were not recognized by the spot localizer due to segmentation failure or elimination. As depicted in Fig. 11e and f, the too small, too large and too elongated spots are not taken into account in the final de-arraying results. This behavior is confirmed by a lower Recall value which measures the sensitivity of the method (R=0.95 in average compared to the perfect score R=1 obtained with Option #3). Although, despite a smaller number of correct spot positions, the estimation of the array coordinate yielded exact results for successfully recognized spots (Precision value P=1 in average) comparing with the ground-truth. In terms of deformation estimation, the estimated potential spot positions provided by the de-arraying with two setting options are almost identical. It thus allows us to localize spots which were not recognized and demonstrates the robustness of our method for estimating the grid deformation.

Regarding the two remaining options (not illustrated in Fig. 11), when both the segmentation and non-linear estimation modules are deactivated (Option #1), ATMAD produced surprisingly very satisfying de-arraying results with a F-score = 0.96 in average on this set of simulated images (see Table 2). This score which is slightly lower than those obtained with Option #4 is due to the monotone and non-oscillating nature of the deformation model used to generate the test images, as described in [22] and illustrated in Fig. 11a and b. Meanwhile, the combination of the segmentation activated and the non-linear estimation deactivated (Option #2) yielded strongly inferior results. The F-score in average is barely 0.91 (corresponding to an accuracy level of 84%). It is mainly due to lower rate of correctly localized spots, implying inaccurate linear estimation for the deformation.

Moreover, we point out that when there is no false positive (i.e. FP = 0 implies P = 1), the Jaccard similarity coefficient (JSC) coincides with the Recall (R) value. This explains why we have obtained the same values for these two performance measures on this set of simulated images. We also observe similar behaviors in some cases on brightfield and fluorescence images when the method tends to eliminate all false detections during the localization step.

### Brightfield images

We have noticed that in the previous experiments with simulated data, our wavelet-based detection algorithm was able to localize all spots on images with constant background. In the case of brightfield TMA images whose background is not constant but generally homogeneous, this approach might still be efficient for spot localization since the situation is much more simpler than in fluorescence imaging. In this section, we focus on the evaluation of ATMAD applied to the ACRS dataset with the Options #3 and #4 (see Table 1) to assess the impact of the ellipse-based segmentation algorithm. In Fig. 12, the de-arraying result with these two setting options on a H&E stained TMA image containing irregularities in the shape of tissue cores is illustrated. The original input image shown in Fig. 12a is the slide cut #9 of the TMA whose the ID is 550-T0011-01 on ACSR’s database. The de-arraying results obtained with the Option #3 and #4 are depicted in Fig. 12c and d respectively. To evaluate the accuracy of these results, we consider in Fig. 12b a reference de-arraying obtained by manual annotation. The latter is presented in the same format (*i.e.* an array representation) as those of the automated de-arraying outcomes to facilitate comparison.

Comparing with an accuracy of 100% obtained on simulated data, localization only based the wavelet method achieved in average approximately 94% of existing TMA cores on ACSR’s data (corresponding to a F-score = 0.96 in average). Indeed, it failed generally to recognize cores with inner hole or cores which are split into parts (see Fig. 12c) since the shape of these cores implies that the wavelet coefficients at their position are lower than the detection threshold – resulting to non detection. Activating the segmentation module does not improve successful recognition rate of the localization step due to the use of detected core position for initializing the ellipse fitting. In our interest, the main role of this module in the localization step is to measure the size and the roundness of detected objects in order to eliminate false detection and to provide reliable input for the estimation of the grid deformation. For this reason, only about 87% of existing cores were correctly recognized during the localization step (corresponding to F-score = 0.91 in average) with the combination of the detection and the segmentation modules due to the segmentation failure and the elimination of outliers. In spite of the difference between the localization results obtained with the deactivation/activation of the segmentation module, the non-linear estimation of the grid deformation using these results however yielded similar de-arraying outcomes as illustrated in Fig. 12c and d. The overall accuracy of the de-arraying procedure with the activation of the ellipse-based segmentation module is approximately 93% (corresponding to F-score = 0.95 in average) compared to 87% (corresponding to F-score = 0.91 in average) if the module is activated (see Table 2). Under the latter setting options, the final recognition rate of tissue cores has increased by about 6% with respect to the rate obtained after the localization step. This improvement is due to the segmentation performed using the potential position which is provided by the estimation of the grid deformation to recognize missed cores during the first step of the de-arraying procedure (for example, some fragmented cores or cores with inner hole were additionally recognized as shown in Fig. 12d in comparison with Fig. 12c). This approach is useful, not only for brightfield images, but also in the case of fluorescence images, in which the contrast between the background and the foreground is often significantly weaker.

For the two remainder options (Options #1 and #2), ATMAD produced slightly inferior scores when compared to those obtained with the Options #3 and #4. It is due to the imprecise estimation of tissue core positions computed with affine registration of the grid. Quantitative similar results were observed in the case of simulated images as reported in Table 2.

### Fluorescence images

In this section, we evaluated ATMAD on a more challenging image dataset which is acquired by fluorescence scanners and characterized by high noise level and non-homogeneous background. Unlike simulated and brightfield images depicting tissues, fluorescence images provided by Innopsys company, are composed of both DNA microarray and TMA images. Examples of DNA and TMA image de-arraying are respectively shown in Figs. 13 and 14.

For the illustrated DNA microarray, we presented in Fig. 11 only the original image, the final de-arraying result and the corresponding manual annotations. Whereas, intermediate results were additionally illustrated in Fig. 12 besides the original image as well as the final result and the ground truth in the case of TMA image to allow step-by-step evaluation.

As expected, the proposed ATMAD method achieved 100% accuracy (corresponding to the perfect F-score = 1) in average on DNA microarray images under all four considered setting options (see Table 2 and Fig. 13). This perfect score was obtained due to the regularity of the size, the shape and the grid of spotted DNA samples which facilitates the localization and the estimation of the array coordinates of each spot.

It is however not possible to reach such performance scores on TMA images in most cases because of the deformation of TMA grid and the irregularities of TMA cores. Indeed, when the segmentation module is deactivated (Options #1 and #3), the localization of TMA cores estimated with only the wavelet-based detection method, often suffers from false positives because erroneous detection of irrelevant objects on the background is not eliminated.

False detection mostly occurs in images with complex background such as those illustrated in Figs. 14a and b. Note that in the case of fluorescence TMA images, the number of false positives is significantly larger than in the case of simulated and brightfield TMA images. On the other hand, tanks to the adaptive threshold derived from the wavelet transform, there is in general no false negative (i.e. all existing tissue cores were detected). These results demonstrate that the detection operation is not too sensitive (perfect recall score R=1 in average), but also it is not precise enough (weak precision score P=0.77 in average) in fluorescence imaging. Consequently, it lowered the overall performance of TMA core localization. In Table 2, the accuracy is only about 79% (corresponding to a *F*-score = 0.87) in average. Note that the linear transform estimation (Option #1) using the set of localizations with false positives, yielded satisfying de-arraying results (with an accuracy of 91% or F-score = 0.94 in average), mainly because robust estimators are used for TMA grid registration. Nevertheless, in some cases the non-linear transform estimation (Option #3) was unable to correctly handle erroneous inputs and to produce reliable de-arraying results.

In order to reduce the number of false positives during the localization step, we combined the wavelet-based detection method with the ellipse-based segmentation method. Despite low-light fluorescence imaging conditions and low contrast in images, the multi-core ellipse-based segmentation perfectly performed with a rate of 100% of successful segmentation over all detected positions. The segmentation procedure provided reliable features of the object found at each detected position (see Fig. 14c). By combining the detection and the segmentation modules, the localizer gave better results; in average, the overall accuracy is about 91% (F-score = 0.94) to be compared to only 79% when the ellipse-based segmentation module is not activated (see Table 2). Given these precise localization results, the non-linear transform estimation produced satisfactory outcomes; the row and column coordinates of most existing TMA cores were accurately computed (Fig. 14f). In average, the de-arraying with activation of both the ellipse-based segmentation and the non-linear transform estimation modules (Option #4) achieved a F-score = 0.96 (corresponding to an accuracy of 92%), which is sightly better than those obtained with Option #1 (F-score = 0.95). We also notice a gain of about 1% in terms of overall accuracy (0.01 in terms of F-score performance) comparing to the localization step. The improvement between the two steps of the de-arraying procedure demonstrates the positive influence of the ellipse-based segmentation module on the overall performance of the proposed ATMAD method.

To sum up, the proposed de-arraying method rarely achieves perfect scores in the case of real (brightfield and fluorescence) images (except those obtained on DNA microarrays) in comparison to simulated images. This weaker performance is often due to the insufficient number of localized cores obtained on images with complex non-homogeneous background and/or highly irregular shapes of tissue cores. Consequently, we get imperfect de-arraying results which represent only array coordinates of each core. In spite of these imperfections, we have noticed that the spline approximation of the grid deformation yields, in most cases, accurate core position. More sophisticated segmentation algorithms can be used to further localize cores which were not recognized, and thus refine de-arraying results.

The majority of the time computing is spent on the detection task to compute the wavelet transform. Overall, the computational cost is less than 5 s for de-arraying a 1000×1000 image. The experiments were performed on a Macbook Pro equipped with 2.7 Ghz Intel Core i7, 16 Gb of RAM and the Mac OS X v. 10.12.4 operating system. The algorithm was implemented in Matlab and we exploited the intrinsic parallelism of the CPU by performing many ellipse-based segmentation in parallel.

## Conclusion

This paper introduced a fast and efficient algorithm for de-arraying TMA by combining wavelet transform, active contour and thin-plate interpolation. The proposed ATMAD algorithm is adapted not only for brightfield images but also for fluorescence images which are more challenging in terms of tissue localization due to complex backgrounds. This difficulty is carried out by a two-step approach: a fast detection followed by a careful segmentation to reduce the number of false alarms. The row and column coordinates of each localized tissue core are next computed by estimating the deformation of the design grid. Using the estimation of the deformation, tissue cores which are missed during localization can be later recognized and it refine thus the de-arraying result.

## Additional file

Additional file 1 Isotropic wavelet frame. Direct wavelet decomposition algorithm and reconstruction. Partial derivatives of the ellipse quadratic form. (PDF 255 kb)

## Abbreviations

DOG: Difference of two Gaussians
FA: False Alarm
HDR: High dynamic range
TMA: Tissue MicroArray
ATMAD: Automatic tissue microarray de-arraying
