# Supplementary material for: ATMAD: robust image analysis for Automatic Tissue MicroArray De-arraying
Source: BMC Bioinformatics. 2018 Apr 19;19:148. doi: 10.1186/s12859-018-2111-8 (PMC5909283; doi:10.1186/s12859-018-2111-8)
Supplement: Supplementary file 1 — Isotropic wavelet frame. Direct wavelet decomposition algorithm and reconstruction. Partial derivatives of the ellipse quadratic form. (PDF 255 kb) [file 12859_2018_2111_MOESM1_ESM.pdf]

METHODOLOGY ARTICLE

ATMAD: robust image analysis for Automatic Tissue MicroArray De-arraying

Hoai Nam Nguyen[1*], Vincent Paveau[2], Cyril Cauchois[2] and Charles Kervrann[1]

*Correspondence:
hoai-nam.nguyen@inria.fr
[1]Inria Rennes - Bretagne Atlantique, Campus universitaire de Beaulieu, 35042 Rennes, France
Full list of author information is available at the end of the article
†Authors' emails:
hoai-nam.nguyen@inria.fr,
v-paveau@innopsys.fr,
cyril.cauchois@innopsys.fr,
charles.kervrann@inria.fr.

Appendix

Isotropic wavelet frame

Mathematically, a wavelet $\psi : \mathbb{R}^d \longrightarrow \mathbb{R}$ is a function of zero average:

$$\int \dots \int_{\mathbb{R}^d} \psi(\mathbf{x})\ d\mathbf{x} \ = \ 0\ , \tag{1}$$

where d is the number of dimensions (usually, $d = 1,\ 2$ or 3). When the wavelet function ψ is isotropic ($i.e.$ $\psi(-\mathbf{x}) = \psi(\mathbf{x}), \forall \mathbf{x} \in \mathbb{R}^d$), we deduce the wavelet atoms $\{\psi_{(\mathbf{t},s)}\}_{(\mathbf{t},s)\in\mathbb{R}^d\times\mathbb{R}_+^*}$ by dilation of ψ with factor s and translation by vector $\mathbf{t}$ as:

$$\psi_{(\mathbf{t},s)}(\mathbf{x}) = \frac{1}{s^d}\ \psi\left(\frac{1}{s}(\mathbf{x}-\mathbf{t})\right)\ . \tag{2}$$

The wavelet transform of a square-integrable function $u \in L^2(\mathbb{R}^d)$ is defined as the inner product (in $\mathbb{R}^d$) of u and the wavelet atom $\psi_{(\mathbf{t},s)}$ as follows:

$$\Psi u(\mathbf{t}, s) = \langle u, \psi_{(\mathbf{t},s)} \rangle = \int \dots \int_{\mathbb{R}^d} u(\mathbf{x})\ \bar{\psi}_{(\mathbf{t},s)}(\mathbf{x})\ d\mathbf{x}\ , \tag{3}$$

where $\bar{\psi}_{(\mathbf{t},s)}$ denotes the complex conjugate of $\psi_{(\mathbf{t},s)}$. To construct a shift invariant representation, we consider the dyadic wavelets derived by discretizing the scale parameter along a dyadic sequence $\{2^j\}_{j\in\mathbb{Z}}$, while the translation parameter is not sampled as:

$$\left\{\psi_j(\mathbf{x}) = \frac{1}{2^{dj}}\ \psi\left(\frac{\mathbf{x}}{2^j}\right)\right\}_{j\in\mathbb{Z}}\ . \tag{4}$$

The family $\{\psi_j\}_{j\in\mathbb{Z}}$ is a frame of $L^2(\mathbb{R}^d)$ according to [2]. Using this family, the isotropic wavelet transform of u at the scale (or resolution) 2^j and the position $\mathbf{x} \in \mathbb{R}^d$ is:

$$\begin{aligned} \Psi_j u(\mathbf{x}) &= \int \dots \int_{\mathbb{R}^d} u(\mathbf{x})\ \frac{1}{2^{dj}}\ \psi\left(\frac{1}{2^j}(\mathbf{x}-\mathbf{t})\right) d\mathbf{t} \\ &= u \star \check{\psi}_j(\mathbf{x}), \end{aligned} \tag{5}$$

where $\check{\psi}_j(\mathbf{x}) = \psi_j(-\mathbf{x}) = \psi_j(\mathbf{x})$ since ψ_j is isotropic. The wavelet coefficient $\Psi_j u(\mathbf{x})$ can also be expressed as the difference of approximations of u at subsequent scales

as follows:

$$\begin{aligned} \Psi_j u(\mathbf{x}) &= u_{j-1}(\mathbf{x}) - u_j(\mathbf{x}) \;, \\ u_j(\mathbf{x}) &= \chi_j \star u_{j-1}(\mathbf{x}) = \lim_{k\to+\infty} (\chi_j \star \ldots \star \chi_{j-k}) \star u(\mathbf{x}), \end{aligned} \tag{6}$$

where u_j and χ_j denote respectively the approximation of u and the smoothing function at the scale 2^j.

The infinite convolution cascade $\phi_j = \lim_{k\to+\infty} (\chi_j \star \ldots \star\chi_{j-k})$ represents the approximation operator applied on u at the given resolution 2^j. In order to favor the simplest computation of the convolution sequence in (7), the selection of $(\chi_j)_{j\in\mathbb{Z}}$ is critical. Among many function families, Gaussian functions are convenient since it satisfies the semi-group property (i.e. the convolution of two Gaussians is also a Gaussian with variance being the sum of the original variances):

$$G_{\sigma_a} \star G_{\sigma_b}(\mathbf{x}) = G_{\sqrt{\sigma_a^2+\sigma_b^2}}(\mathbf{x}) \;, \tag{7}$$

where G_σ is a Gaussian with standard deviation σ.

Therefore, if we choose χ_j being a Gaussian G_{σ_j} with standard deviation σ_j, the convolution sequence $(\chi_j \star \ldots \star \chi_{j-k})$ can be easily computed by summing $\sigma_j^2, \ldots, \sigma_{j-k}^2$. Since resolution decreases by a factor 2 between two consecutive scales, it is appropriate to set $\sigma_j = 2\sigma_{j-1} = 2^{j-1}\sigma_1$, where σ_1 is a reference standard deviation according to the Shannon-Nyquist sampling step. Thus, we have:

$$\sum_{k=0}^{+\infty} \sigma_{j-k}^2 = \sum_{k=0}^{+\infty} \left(2^{j-k-1}\sigma_1\right)^2 = \frac{4^j}{3}\sigma_1^2 \;. \tag{8}$$

Then, the approximation operator at the scale 2^j can be expressed as a convolution with a Gaussian of variance $(4^j/3)\sigma_1^2$:

$$\phi_j(\mathbf{x}) = \lim_{k\to+\infty} (\chi_j \star \ldots \star \chi_{j-k})(\mathbf{x}) = G_{\sqrt{(4^j/3)\sigma_1^2}}(\mathbf{x}) \;. \tag{9}$$

Additionally, from equations (5) to (7), we deduce:

$$\psi(\mathbf{x}) = 2^{dj}(\phi_{j-1}(2^j\mathbf{x}) - \phi_j(2^j\mathbf{x})) = \phi_{-1}(\mathbf{x}) - \phi_0(\mathbf{x}) \;. \tag{10}$$

By generalizing the equation expressed the relation of the mother wavelet ψ and its associate scaling function ϕ as described in [3] as follows:

$$\frac{1}{2^d}\psi\left(\frac{\mathbf{x}}{2}\right) = \phi(\mathbf{x}) - \frac{1}{2^d}\phi\left(\frac{\mathbf{x}}{2}\right), \tag{11}$$

a closed-form solution of the scaling function ϕ (also known as father wavelet) is obtained as:

$$\phi(\mathbf{x}) = \phi_0(\mathbf{x}) = G_{\sigma_1/\sqrt{3}}(\mathbf{x}) = \frac{1}{(2\pi\sigma_1^2/3)^{d/2}} \exp\left(-\frac{3\|\mathbf{x}\|_2^2}{2\sigma_1^2}\right). \tag{12}$$

Since ϕ is Gaussian, ψ is defined as the difference of two Gaussians (DOG) with the first standard deviation equal to half of the second one. Given a value of σ_1 as input, the wavelet atoms ψ_j which are also DOGs are determined using the equations (4) and (10). Hence, a class of isotropic wavelets can be simply characterized by only one parameter.

Direct wavelet decomposition algorithm and reconstruction

Starck *et al.* proposed a method for performing the isotropic undecimated wavelet transform in [4]. This algorithm is called the Starlet transform. Like the standard undecimated wavelet transforms, the Starlet transform uses the *à trous* algorithm [1] which inserts zeros (holes) in the discrete convolution kernel at each iteration. This kernel is a low-pass filter derived from the normalized cardinal B-spline of order 3:

$$\begin{aligned} h^{(1D)}[k] &= \frac{1}{16}[1,\ 4,\ 6,\ 4,\ 1], \quad k \in \{-2,-1,0,1,2\}, \\ B_3(u) &= \frac{1}{12}(|u-2|^3 - 4|u-1|^3 + 6|u|^3 \\ &\qquad - 4|u+1|^3 + |u+2|^3). \end{aligned}$$

Yet, as the order tends to infinite, the normalized cardinal B-spline tend to a Gaussian function The kernel used for wavelet decomposition is then an approximation of a Gaussian. To avoid the decimation at each level, the insertion of holes in the kernel leads to approximation errors, since the kernels with holes are far from their corresponding scaling functions. The error cumulation through iterations increases with the level of the decomposition. In addition, the iterative convolution process involves the dependence of the higher level of the decomposition on its previous levels. To sum up, the limits of *à trous* algorithm are its inaccuracy and computational cost.

To overcome these limits, the discrete wavelet decomposition of a function u at the desired scale 2^j can be obtained by performing the discrete convolution between u and the corresponding wavelet atom ψ_j. In practice, since all computations are performed over discrete signals, the input signal is not u but its discrete approximation counterpart. Without losing the generality, one can assume this approximation is u_0. In fact, using (6) and (7), we have:

$$\begin{aligned} \Psi_j u(\mathbf{x}) &= u_{j-1}(\mathbf{x}) - u_j(\mathbf{x}) \\ &= \begin{cases} (\chi_{j-1} * \ldots \star \chi_1 - \chi_j \star \ldots \star \chi_1) \star u_0(\mathbf{x}) \text{ if } j > 1 \\ u_0(\mathbf{x}) - \chi_1 \star u_0(\mathbf{x}) \text{ if } j = 1 \end{cases} \\ &= (\phi^0_{j-1} - \phi^0_j) \star u_0(\mathbf{x}) \\ &= \psi^0_j \star u_0(\mathbf{x}), \end{aligned} \tag{13}$$

$$\text{where} \quad \phi^0_j = \begin{cases} \chi_j \star \ldots \star \chi_1 & \text{if } j > 1, \\ \delta & \text{if } j = 1. \end{cases}$$

The key idea of our method is the computation of the scaling function ϕ^0_j which results the convolution sequence $\chi_j \star \ldots \star \chi_1$. Since χ_j is chosen as a Gaussian with

**Level** (j)	**Std. of** χ_j (σ_j)	**Variance of** ϕ_j^0 (v_j^2)
1	σ_1	σ_1^2
2	$2\sigma_1$	$5\sigma_1^2$
3	$4\sigma_1$	$21\sigma_1^2$
4	$8\sigma_1$	$85\sigma_1^2$
5	$16\sigma_1$	$341\sigma_1^2$
6	$32\sigma_1$	$1365\sigma_1^2$

Table 1 Computation of the variance of the scaling function ϕ_j^0 at first few levels.

variance $\sigma_j^2 = 4^{j-1}\sigma_1^2$, the function of interest ϕ_j^0 is also a Gaussian with variance:

$$v_j^2 = \begin{cases} \sum_{k=1}^{j} 4^{k-1}\sigma_1^2 & \text{if } j \geq 1, \\ 0 & \text{if } j = 0. \end{cases} \tag{14}$$

The values of v_j^2 at first few levels are reported in the Table 1.

Hence, there are two ways to compute $\Psi_j u$. The fastest way is to compute directly the wavelet atom $\psi_j^0(\mathbf{x}) = \phi_{j-1}^0(\mathbf{x}) - \phi_j^0(\mathbf{x}) = G_{v_{j-1}}(\mathbf{x}) - G_{v_j}(\mathbf{x})$ (with convention $G_0(\mathbf{x}) = \delta(\mathbf{x})$). Then, the wavelet decomposition map is the convolution product $\Psi_j u(\mathbf{x}) = \psi_j^0 \star u_0(\mathbf{x})$. The alternative way is to compute two convolutions $u_{j-1}(\mathbf{x}) = \phi_{j-1}^0 \star u_0(\mathbf{x})$ and $u_j(\mathbf{x}) = \phi_j^0 \star u_0(\mathbf{x})$, thus the wavelet decomposition is derived by taking the difference of two computed results. This way is slower than the first one but it is useful to reconstruct the input signal u_0.

By construction, for any given level j, the formula of reconstruction of u_0 is:

$$\begin{aligned} u_0(\mathbf{x}) &= \sum_{k=1}^{j} \Psi u_k(\mathbf{x}) + u_j(\mathbf{x}) \qquad (15) \\ &= \sum_{k=1}^{j-1} (u_{k-1} - u_k)(\mathbf{x}) + \Psi_j u(\mathbf{x}) + u_j(\mathbf{x}) \\ &= u_0(\mathbf{x}) - u_{j-1}(\mathbf{x}) + \Psi_j u(\mathbf{x}) + u_j(\mathbf{x}). \end{aligned}$$

The perfect reconstruction of u_0 is therefore possible if $u_0 - u_{j-1}$ and u_j are known. Yet, u_{j-1} and u_j are computed by using the alternative way of the wavelet decomposition. Instead of returning only the wavelet decomposition $\Psi_j u$, $u_0 - u_{j-1}$ and u_j are also stored if the user needs to perform the reconstruction step. This approach can be extended for multi-scale reconstruction. In fact, if we have $(\Psi_j u)_{j \in J}$ where $J = \{j_1, j_2, \ldots, j_{|J|}\} \subset \mathbb{N}^*$, the reconstruction formula is written as:

$$u_0(\mathbf{x}) = \frac{1}{|J|} \sum_{k=1}^{|J|} (u_0 - u_{j_k - 1} + \Psi u_{j_k} + u_{j_k})(\mathbf{x}). \tag{16}$$

In practice, wavelet-based algorithms compute over a set of subsequent scales (i.e. $J = \{j_0, j_0{+}1, \ldots, j_0{+}|J|{-}1\}$). In this case, the reconstruction formula is therefore:

$$u_0(\mathbf{x}) = u_0(\mathbf{x}) - u_{j_0 - 1}(\mathbf{x}) + \sum_{k=0}^{|J|-1} \Psi u_{j_0 + k}(\mathbf{x}) + u_{j_0 + |J| - 1}(\mathbf{x}). \tag{17}$$

Partial derivatives of the ellipse quadratic form

Let $\{\mathbf{x}_0, a, b, \theta\}$ be the parameters of the ellipse Γ where $\mathbf{x}_0 = (x_0, y_0)$ is the center, a and b are the semi major and minor axes, and θ is the angle of rotation. We consider the quadratic form induced by Γ as:

$$\begin{aligned}
\|\mathbf{x}-\mathbf{x}_0\|_\Gamma^2 &= \left\| \begin{bmatrix} a^{-1} & 0 \\ 0 & b^{-1} \end{bmatrix} \begin{bmatrix} \cos\theta & \sin\theta \\ -\sin\theta & \cos\theta \end{bmatrix} (\mathbf{x}-\mathbf{x}_0) \right\|_2^2 \\
&= \left\| \begin{bmatrix} \dfrac{\cos\theta}{a} & \dfrac{\sin\theta}{a} \\ -\dfrac{\sin\theta}{b} & \dfrac{\cos\theta}{b} \end{bmatrix} (\mathbf{x}-\mathbf{x}_0) \right\|_2^2 \\
&= \left(\frac{\cos\theta}{a}(x-x_0) + \frac{\sin\theta}{a}(y-y_0) \right)^2 \\
&\quad + \left(-\frac{\sin\theta}{b}(x-x_0) + \frac{\cos\theta}{b}(y-y_0) \right)^2 .
\end{aligned} \tag{18}$$

The partial derivatives of $\|\mathbf{x}-\mathbf{x}_0\|_\Gamma^2$ with respect to $\{\mathbf{x}_0, a, b, \theta\}$ are given by:

$$\begin{aligned}
\frac{\partial \|\mathbf{x}-\mathbf{x}_0\|_\Gamma^2}{\partial x_0} &= -2 \left[\frac{\cos\theta}{a} \left(\frac{\cos\theta}{a}(x-x_0) + \frac{\sin\theta}{a}(y-y_0) \right) \right. \\
&\quad \left. - \frac{\sin\theta}{b} \left(-\frac{\sin\theta}{b}(x-x_0) + \frac{\cos\theta}{b}(y-y_0) \right) \right]
\end{aligned} \tag{19}$$

$$\begin{aligned}
\frac{\partial \|\mathbf{x}-\mathbf{x}_0\|_\Gamma^2}{\partial y_0} &= -2 \left[\frac{\sin\theta}{a} \left(\frac{\cos\theta}{a}(x-x_0) + \frac{\sin\theta}{a}(y-y_0) \right) \right. \\
&\quad \left. + \frac{\cos\theta}{b} \left(-\frac{\sin\theta}{b}(x-x_0) + \frac{\cos\theta}{b}(y-y_0) \right) \right]
\end{aligned} \tag{20}$$

$$\frac{\partial \|\mathbf{x}-\mathbf{x}_0\|_\Gamma^2}{\partial a} = -\frac{1}{a} \left(\frac{\cos\theta}{a}(x-x_0) + \frac{\sin\theta}{a}(y-y_0) \right)^2 \tag{21}$$

$$\frac{\partial \|\mathbf{x}-\mathbf{x}_0\|_\Gamma^2}{\partial b} = -\frac{1}{b} \left(-\frac{\sin\theta}{b}(x-x_0) + \frac{\cos\theta}{b}(y-y_0) \right)^2 \tag{22}$$

$$\begin{aligned}
\frac{\partial \|\mathbf{x}-\mathbf{x}_0\|_\Gamma^2}{\partial \theta} &= 2 \left(\frac{b}{a} - \frac{a}{b} \right) \left(\frac{\cos\theta}{a}(x-x_0) + \frac{\sin\theta}{a}(y-y_0) \right) \\
&\quad \times \left(-\frac{\sin\theta}{b}(x-x_0) + \frac{\cos\theta}{b}(y-y_0) \right) .
\end{aligned} \tag{23}$$

Author details
[1]Inria Rennes - Bretagne Atlantique, Campus universitaire de Beaulieu, 35042 Rennes, France. [2]Innopsys, Parc d'Activités Activestre, 31390 Carbonne, France.

References

1. Starck, J.L., Murtagh, R.: Image restoration with noise suppression using wavelet transform. Astronomy and Astrophysics **288**, 342–348 (1994)
2. Mallat, S.: A Wavelet Tour of Signal Processing (third Edition: The Sparse Way). Academic Press, Boston (2008)
3. Starck, J.-L., Murtagh, F.: Astronomical Image and Data Analysis. Springer, 978-3-540-33025-7 (2002)
4. Starck, J.-L., Fadili, J., Murtagh, F.: The undecimated wavelet decomposition and its reconstruction. IEEE Trans Image Processing **16**(2), 297–309 (2007)
